# Serum cytokine patterns are modulated in infants fed formula with probiotics or milk fat globule membranes: A randomized controlled trial

**DOI:** 10.1371/journal.pone.0251293

**Published:** 2021-05-13

**Authors:** Xiaonan Li, Yongmei Peng, Zailing Li, Britt Christensen, Anne B. Heckmann, Carina Lagerqvist, Hans Stenlund, Bo Lönnerdal, Olle Hernell, Christina E. West

**Affiliations:** 1 Department of Child Health Care, Childrens Hospital of Nanjing Medical University, Nanjing, China; 2 Department of Children Health Care, Childrens Hospital of Fudan University, Shanghai, China; 3 Department of Pediatrics, Beijing University Third Hospital, Bejing, China; 4 Arla Innovation Center, Arla Foods amba, Skejby, Denmark; 5 Arla Foods Ingredients Group P/S, Viby, Denmark; 6 Department of Clinical Sciences, Pediatrics, Umeå University, Umeå, Sweden; 7 Departments of Public Health and Clinical Medicine, Epidemiology and Global Health, Umeå University, Umeå, Sweden; 8 Department of Nutrition, University of California, Davis, California, United States of America; PLOS, UNITED KINGDOM

## Abstract

**Background:**

Proteins and lipids of milk fat globule membrane (MFGM) and probiotics are immunomodulatory. We hypothesized that *Lactobacillus paracasei* ssp. *paracasei* strain F19 (F19) would augment vaccine antibody and T helper 1 type immune responses whereas MFGM would produce an immune response closer to that of breastfed (BF) infants.

**Objective:**

To compare the effects of supplementing formula with F19 or bovine MFGM on serum cytokine and vaccine responses of formula-fed (FF) and BF infants.

**Design:**

FF infants were randomized to formula with F19 (n = 195) or MFGM (n = 192), or standard formula (SF) (n = 194) from age 21±7 days until 4 months. A BF group served as reference (n = 208). We analyzed seven cytokines (n = 398) in serum at age 4 months using magnetic bead-based multiplex technology. Using ELISA, we analyzed anti-diphtheria IgG (n = 258) and anti-poliovirus IgG (n = 309) concentrations in serum before and after the second and third immunization, respectively.

**Results:**

Compared with SF, the F19 group had greater IL-2 and lower IFN-γ concentrations (p<0.05, average effect size 0.14 and 0.39). Compared with BF, the F19 group had greater IL-2, IL-4 and IL-17A concentrations (p<0.05, average effect size 0.42, 0.34 and 0.26, respectively). The MFGM group had lower IL-2 and IL-17A concentrations compared with SF (p<0.05, average effect size 0.34 and 0.31). Cytokine concentrations were comparable among the MFGM and BF groups. Vaccine responses were comparable among the formula groups.

**Conclusions:**

Contrary to previous studies F19 increased IL-2 and lowered IFN-γ production, suggesting that the response to probiotics differs across populations. The cytokine profile of the MFGM group approached that of BF infants, and may be associated with the previous finding that infectious outcomes for the MFGM group in this cohort were closer to those of BF infants, as opposed to the SF group. These immunomodulatory effects support future clinical evaluation of infant formula with F19 or MFGM.

## Introduction

Human milk is plentiful in bioactive components with non-nutritive physiological activities with potential to favorably modulate immune ontogeny in infancy [1, 2]. Milk fat globules in human milk are encircled by a membrane system with an inner monolayer, which originates from the endoplasmic reticulum of the mammary cells and an outer bilayer originating from the apical membrane of epithelial cells of the mammary gland during lactation [1]. The milk fat globule membrane (MFGM) thus contains various cellular components e.g. cholesterol, glycerophospholipids, sphingolipids and proteins, some of which are glycosylated. Among the latter, the proteins mucin 1, lactadherin and butyrophilin are present in high concentrations in MFGM, and have been reported to influence adaptive immunity [3–5]. This has led to emerging interest in the immunomodulatory role of MFGM proteins and lipids.

Traditionally, infant formulas are manufactured from skim milk powder and whey protein concentrate while the milk fat is removed and replaced by a mix of vegetable oils. Consequently, standard infant formula contains less bioactive MFGM proteins and lipids than human milk. Bovine milk fractions enriched in MFGM are now marketed. Although the commercial bovine MFGM concentrate Lacprodan® MFGM-10 is not identical to human MFGM, nor as dynamic as is the case for human and bovine milk MFGM, it can be added to infant formula to support the development of the immune system [6, 7]. We have shown that the addition of bovine MFGM concentrate to infant formula is safe in terms of infant growth and adverse effects [8–10]. In one of our studies [9], we also studied the effects on MFGM on the incidence of acute otitis media. In that study, infants fed the MFGM supplemented formula had lower incidence of acute otitis media than infants fed standard formula (SF), which was associated with lower abundance of the common otopathogen *Moraxella catharralis* in the oral cavity [11].

The gradual colonization of the mucosal surfaces of the oral cavity and the gut parallels the ontogeny of both the innate and adaptive immune systems [12–16]. It has been theorized that the addition of probiotics in infant formula or as a supplement will supply microbial stimulation, thereby aiding the establishment of appropriate T regulatory and T helper (Th) 1 type responses [17]. We previously reported that feeding the probiotic *Lactobacillus paracasei* ssp. *paracasei* F19 (F19) during weaning from breastfeeding promoted Th 1 type and Th 17 type maturation during the second half of infancy and augmented the adaptive immune response to diphtheria vaccine [18–20]. The safety of feeding F19 to infants as judged by growth and lack of adverse effects has also been demonstrated in previous studies [10, 21–23].

The present study builds on a previous study of this group [10] in which healthy infants were randomized to three different formula groups; formula with added F19 or MFGM, or SF. A breastfed group (BF) served as reference. The primary outcome of the study was infectious episodes, which were similar in all formula groups during the intervention. However, compared to the BF group, the SF group but not the F19 or MFGM group had more days and episodes of fever [10]. In addition, all infectious outcomes for the MFGM group were similar to the BF group. As infant formula with added bioactive components has potential to influence the development of the adaptive immune system [1, 2, 24, 25], we hypothesized that addition of the probiotic F19 would augment vaccine antibody responses and promote a Th1 type immune response while MFGM would produce an immune response closer to the immune profile of BF infants. Here we report on the secondary outcomes: vaccine antibody responses, cytokine patterns and eczema.

## Methods

### Study design

This was a multicenter trial, registered at www.clinicaltrials.gov (NCT01755481) and performed at study centers in China: Nanjing (Childrens’ Hospital of Nanjing Medical University, Nanjing Maternity and Child Health Care Hospital, the Second Affiliated Hospital of Nanjing Medical University, Nanjing Secondary Hospital and Huaian Maternity and Child Health Hospital); Shanghai (Childrens’ Hospital of Fudan University, Clinical Center for Public Health of Fudan University); Beijing (Peking University Third Hospital, Beijing Ditan Hospital Capital Medical University and The First Hospital of Jilin University) as previously reported [10]. The study was approved by the IRB at University of California Davis, USA, the Regional Ethical Review Boards in Nanjing, Shanghai and Beijing, China, and performed according to the principles of the Declaration of Helsinki. Study staff gave oral and written information to parents/caregivers. Written consent was obtained from the parents or caregivers of all infants before inclusion in the study [10].

### Inclusion criteria and background characteristics

As previously described [10], this was a randomized, double-blind, controlled trial investigating three different infant formulas. A BF reference group was also included. In brief, infants were recruited from December 2013 to August 2016. Inclusion criteria were infants born full term (gestational age at birth 37 to 42 weeks), birth weight >2500 g and <4000 g, absence of chronic disease, parent(s) or legal representative should speak and comprehend Chinese. Exclusion criteria were: any malformation, handicap or congenital disease that might impair normal infant feeding or growth, antibiotic treatment or previous consumption of infant formula with prebiotics and/or probiotics. Additional inclusion criteria for the FF groups were: healthy infants of mothers who were not able to breastfeed, or voluntarily abstained from breastfeeding at inclusion (infant age of 21±7 days). Exclusion criteria for the FF groups were: any breastfeeding at the age of 28 days. Additional inclusion criteria for the BF group were: exclusive breastfeeding from birth and mothers intending to breastfeed >80% for at least 5 months. Exclusion criteria for the BF group: infants that consumed >20% infant formula of their estimated total intake at age 28 days. We collected information on background characteristics at the time of recruitment and information on birth weight, dietary patterns and parental education for all excluded and drop-out infants [10].

### Infant formulas

All study formulas were produced from bovine milk powder by Arla Foods amba, Denmark. The added probiotic *L*. *paracasei* ssp. *paracasei* strain F19 was from Chr. Hansen, Denmark and Lacprodan^®^ MFGM-10 from Arla Foods Ingredients group P/S, Denmark. The complete composition of the three formulas has been reported previously [10] and the final study formulas were manufactured in Hohhot, China according to Chinese regulations in a strict hygienic setting, conforming to all requirements for human consumption.

### Randomization and intervention groups

Infants in the FF groups were randomly assigned to one of the three infant formulas; SF, the same formula with the addition of *L*. *paracasei* ssp. *paracasei* strain F19 at a dose of 10^8^ CFU per L, (F19) or Lacprodan^®^ MFGM-10 (27.5 g of total protein per kg powder, or 5 g per L prepared formula) (MFGM). The intervention lasted from inclusion at 21±7days until the end of the 4th month. An independent statistician generated the randomization list and as described [10], we used a computerized randomization tool in blocks of 24, stratified for sex (12 boys and 12 girls) and type of formula using color-coding (8 of each color). A block size of 8 (4 boys and 4 girls) was used in the BF group. The infant formulas were identical in smell and taste, and were dispensed in identical boxes marked with a color code number, together with detailed instructions on preparation of the formula. Before the start of the intervention, infants were fed SF if FF had been initiated. Starting at the 5^th^ month to the end of the 6^th^ month of age, all infants in the formula groups were fed SF. If breast milk supply was not sufficient, BF infants were fed SF although not exceeding 20% of their estimated total intake based on a three-day formula intake record [10]. Study staff enrolled participants and assigned them to the intervention. Both parents and staff were blinded until analyses had been finalized. Complementary feeding was not permitted during the intervention but was introduced no later than at 26 weeks of age in accordance with current guidelines. Vitamin D supplementation was given following current guidelines. Parents/caretakers whose infants dropped out were asked to remain in the study for follow-up on an intention-to-treat (ITT) basis [10].

### Study visits, immunization schedule and blood samplings

The families visited the study clinics at baseline (inclusion) and then at 1, 2, 3, 4, 5, 6, 9 and 12 months of age [10]. At 12 months of age, a study doctor performed an evaluation of the presence of eczema based on Scoring Atopic Dermatitis (SCORAD) [26]. Data on growth and adverse effects were also collected at the study visits, and have been reported [10].

All infants were immunized at their local health care clinic. Infants received oral poliovirus (OPV) vaccination at 2, 3 and 4 months and were also immunized against diphtheria- and tetanus toxoid and acellular pertussis (DTaP) at 3, 4 and 5 months of age following the national immunization program. Due to regional differences and parental request, some infants were instead immunized with DTaP, polio and *Haemophilus influenzae* type b at 2, 3 and 4 months of age. In order to assess the anti-diphtheria and anti-poliovirus IgG responses in serum following the second and third vaccine dose, respectively, venous blood (0.5 to 2 ml) was collected by trained study staff before and then 4 weeks after the second immunization to diphtheria and the third immunization to OPV. Thus, for anti-poliovirus IgG analyses, only infants that had received OPV were included. Following centrifugation at 1300 x g for 10 min, serum was collected and immediately frozen at -80°C until shipped on dry ice to the sites of analysis.

### Cytokine and vaccine responses

Serum samples were thawed, shaken and centrifuged at 10 000 x g for 10 min before cytokine levels were assayed in duplicate according to the manufacturers’ instructions. Concentrations of IL-2, IL-4, IL-6, IL-17A, IFN-γ and TNF-α were measured using Milliplex Human High Sensitivity T Cell Magnetic Bead 6-plex Panel (HSTCMAG-28SK-06; EMD Millipore; Merck KGaA, Germany) and TGF-β1 using a TGF-β1 Magnetic Bead single-plex Kit (TGFBMAG-64K-01; EMD Millipore; Merck KGaA, Germany). All cytokine measurements were carried out using a Bio-Plex 200 instrument (Bio-Rad Laboratories, Hercules, CA). Concentrations of IL-2, IL-4, IL-6, IL-17A, IFN-γ and TNF-α were read from a 7-point calibration curve and TGF-β1 from a 6-point calibration curve, and calculations were made with Bio-Plex Manager 6.2 (Bio-Rad Laboratories, Hercules, CA). The inter-assay coefficient of variation, for all cytokines measured, was <12%.

For quantitative analysis of anti-diphtheria- and anti-poliovirus-IgG antibodies in human serum, ELISA kits based on the sandwich principle were purchased from Immuno-Biological Laboratories (IBL) Co., Ltd, Tokyo, Japan. Prior to use, both assays were validated by Triskelion B.V. (Zeist, The Netherlands). For validation, standard samples as included in the commercially available ELISA kits for diphtheria toxin and poliovirus, respectively, were used for the composition of calibration lines and QC samples. In each of the ELISA kits for diphtheria (IBL product code RE56191) and poliovirus (IBL product code RE56921), 4 positive control standard samples are included. For both types of ELISAs, calibration lines were composed of 7 concentration points. Besides for the generation of calibration lines, these control samples were used for the preparation of QC samples.

## Statistical methods

Calculation on sample power was done for the primary outcome infectious episodes. As previously described [10], a sample size of 540 infants (180 in each group) was needed to detect a difference of 20% in the incidence of infectious episodes with 80% power (5% significance). We aimed at recruiting 800 infants to allow for a drop-out rate of 15–20%. Data collection and analysis adhered to the project plan, which is available as supplemental information, however, the current study was based on the availability of blood samples. Data were collected on case report forms and were manually entered into EpiData by two independent persons. If any discrepancies between the data entered, this was flagged by the system and data entry was checked again. The database was locked before final analyses. Comparison of demographical characteristics between the four groups was done using ANOVA for quantitative variables and chi square test for categorical variables. Cytokine concentrations that were out of range were replaced by the minimal detectable concentration; these values were provided by the manufacturer. For comparison of cytokine and vaccine concentrations the three FF groups were compared and then a comparison between the FF groups and BF group were done. Comparison of cytokine and vaccine concentrations between the FF groups were done with Kruskal-Wallis test and when relevant, pairwise comparisons were done according to the Bonferroni procedure. For effect size measures, Cohen’s d was calculated by dividing the differences between means by the pooled standard deviation All calculations were done using SPSS v 23 (IBM SPSS Statistics, Armonk, NY). The significance level was set to 5%.

## Results

### Study subjects and demographic characteristics

**Fig 1:**
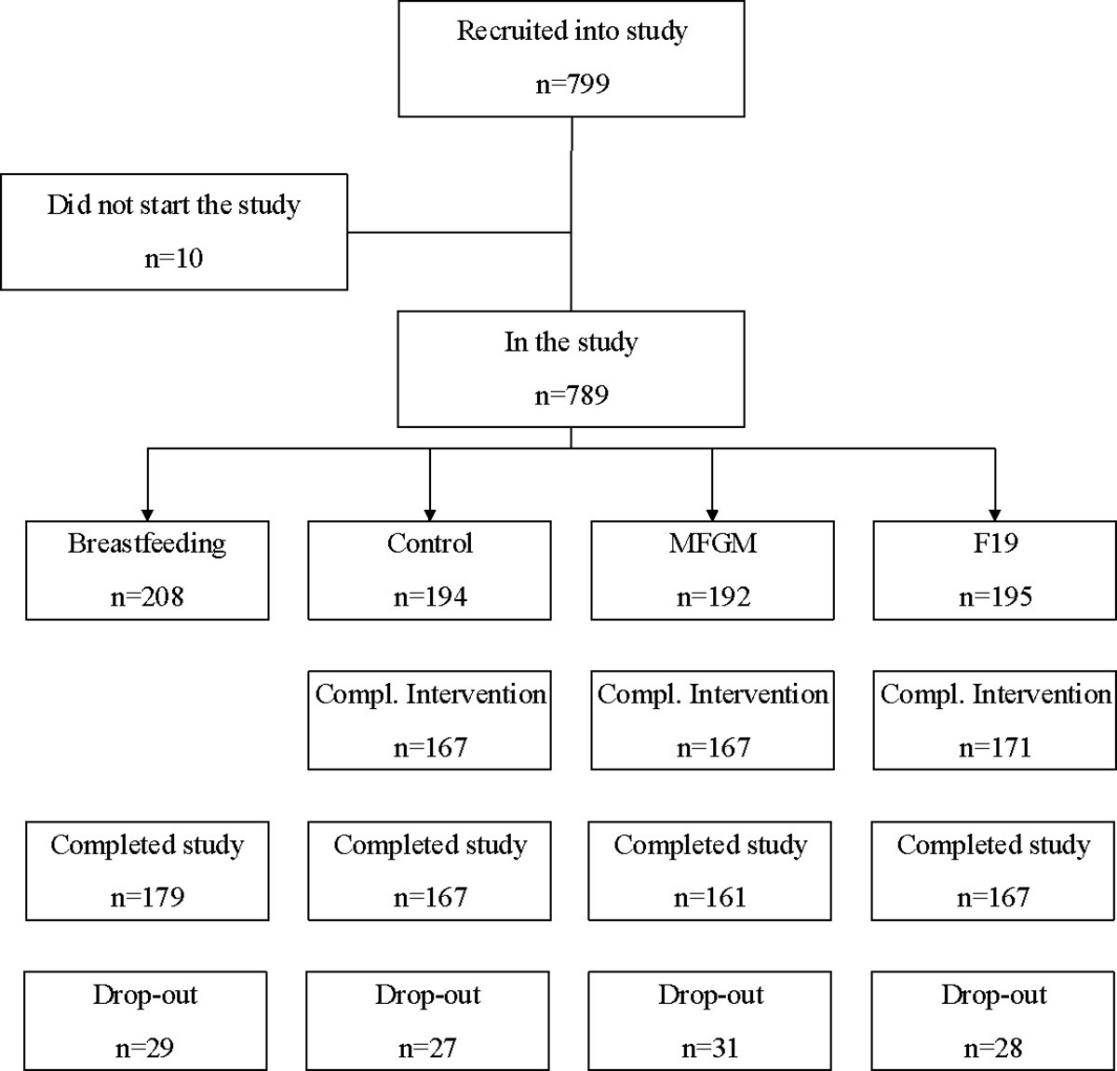
Study flow chart. The figure was previously published in “Feeding infants formula with probiotics or milk fat globule membrane: A double-blind randomized controlled trial”. Li, et al. Front Pediatr 2019; 7:347.

Recruitment and drop-out rates are displayed in Fig 1. There were no differences in demographic characteristics among the FF groups. However, gestational age (p = 0.02), parental education level (p<0.001) and proportion of no siblings (p = 0.005) were lower for the formula-fed groups combined than for the BF group (Table 1).

**Table 1:** Demographic characteristics of the formula-fed and breastfed groups.

Variables	BF (n = 98)	SF (n = 99)	MFGM (n = 100)	F19 (n = 101)
Birth weight (g), mean (sd)	3385 (334)	3279 (376)	3282 (420)	3301 (418)
Sex, girls (%)	51.09	51.5	56.0	46.9
Gestational age (weeks)	39.0	38.6	38.8	38.8
Siblings (% with no siblings)	81.1	64.6	67.0	66.3
Delivery (% caesarean)	43.9	59.6	62.8	52.5
Pregnancy complications (%)	7.1	6.1	10.0	5.0
Maternal age (years), mean (sd)	29.4 (3.7)	29.6 (3.9)	28.9 (4.4)	29.2 (4.2)
Paternal age (years), mean (sd)	31.3 (4.1)	30.9 (4.2)	31.1 (4.9)	31.2 (5.1)
Maternal education (%)	2.0/26.5/71.4	12.1/50.5/36.4	9.0/46.0/44.0	13.9/32.7/53.9
Paternal education (%)	2.0/21.4/76.5	13.1/48.5/38.4	7.0/46.0/45.0	10.0/39.6/48.5

*There are no statistically significant differences between treatment groups for any variable.

BF; breast-fed reference group, SF; standard formula, MFGM; formula supplemented with milk fat globule membrane, F19; formula supplemented with *L*. *paracasei* ssp. *paracasei* strain F19.

Education (%) refers to low/middle/high education level, i.e., <12 y/< 17 y/ > 20 y of school and university education.

### Assessment of eczema at the clinical study visit

At age 12 months, a single child had diagnosed eczema in the F19 group, and the corresponding numbers in the MFGM and SF groups were 2 and 4, respectively. No child had eczema in the BF group. A single child in the MFGM group had an objective score >40, thereby indicating severe eczema, whereas the other children had an objective score of <40, indicating mild to moderate eczema.

### Cytokine responses

We analyzed a panel of cytokines at 4 months of age using magnetic bead-based multiplex technology. The following marker cytokines were analyzed; IL-2 (general T cell stimulation), IFN-γ (Th1 type response), IL-4 (Th2 type response), IL-6 and TNF-α (inflammatory response), IL-17A (Th17 type response) and TGF-β1 (T regulatory response).

**Fig 2:**
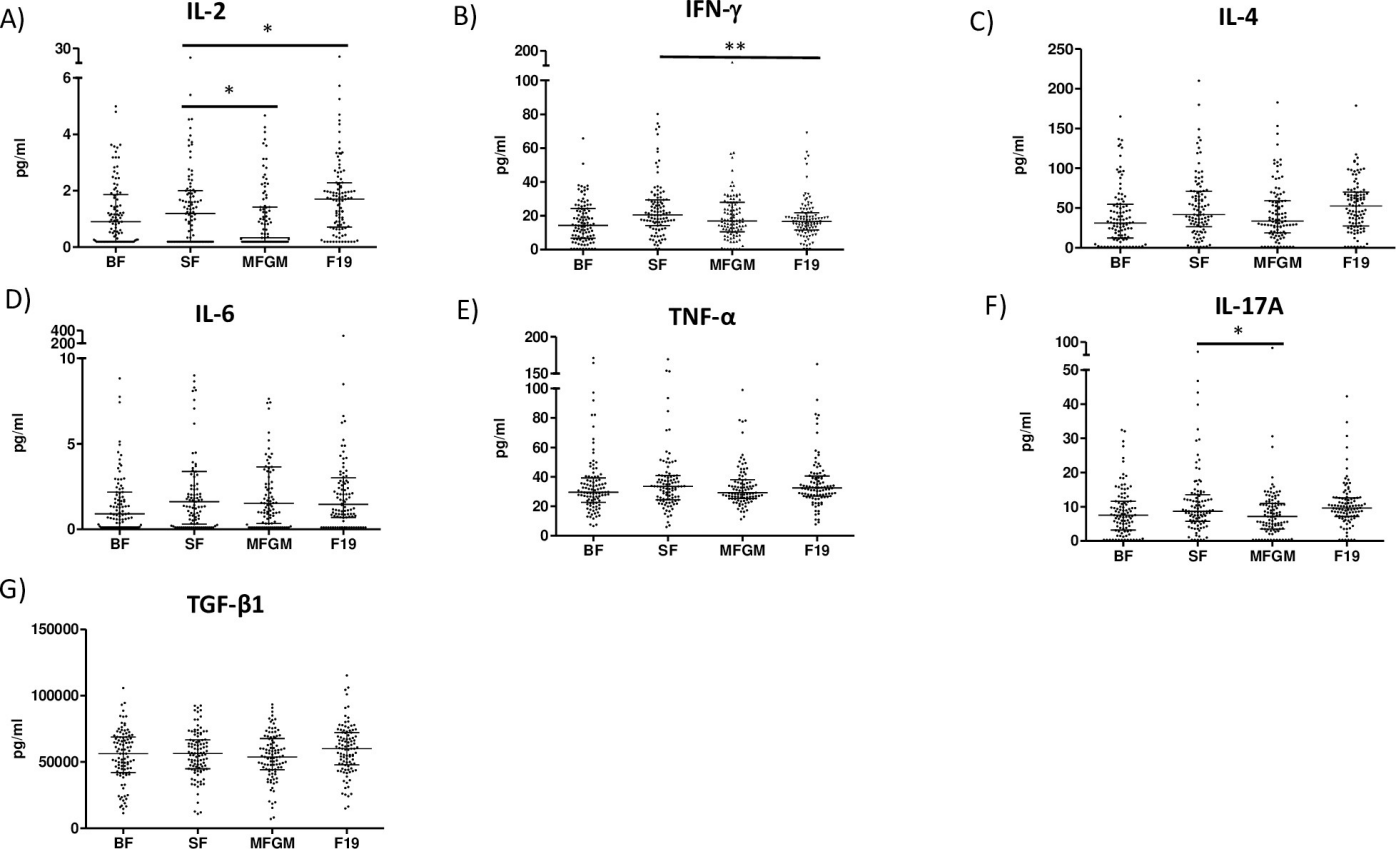
Cytokine responses. At 4 months of age cytokine concentrations were analyzed in 98 infants in the breastfed (BF) group, 99 infants in the standard formula (SF) group, 99 infants in the milk fat globule membrane (MFGM) group and 100 infants in the *Lactobacillus* F19 (F19) group. The MFGM group had lower IL-2 (p = 0.028, effect size 0.34) and IL-17A concentrations (p = 0.014, effect size 0.31) compared with SF. IL-2 concentrations were greater in the F19 compared with the SF group (p = 0.024, effect size 0.14) whereas the IFN-γ concentrations were lower (p = 0.008, effect size 0.39). Scatterplots show median, 25^th^ and 75^th^ percentile. *p<0.05 **p<0.005.

In comparison with the SF group, the F19 group had greater IL-2 concentrations (p = 0.024, effect size 0.14) whereas the IFN-γ concentrations were lower (p = 0.008, effect size 0.39) (Fig 2A and 2B). There were no other differences in cytokine concentrations when comparing the SF and F19 groups. IL-2 (p<0.001, effect size 0.42), IL-4 (p = 0.003, effect size 0.34) and IL-17A (p = 0.033, effect size 0.26) concentrations were higher in the F19 group compared with the BF reference group (Fig 2A, 2C and 2F).

Compared with SF, the MFGM group had lower IL-2 (p = 0.028, effect size 0.34) and IL-17A concentrations (p = 0.014, effect size 0.31) suggesting lower general T cell and Th 17 activation (Fig 2A and 2F). The concentrations of all cytokines were comparable in the MFGM and the BF reference groups (p>0.05) (Fig 2A–2G).

### Vaccine responses

We assessed IgG antibody concentrations in serum following immunization to diphtheria and OPV. All infants had received two doses of diphtheria and three doses of OPV. As displayed in Table 2, there were no differences in anti-diphtheria nor anti-poliovirus IgG concentrations in serum between the SF and F19 groups, nor between the SF and MFGM groups (p>0.05). In comparison with the BF reference group, the formula groups combined had higher serum IgG antibody concentrations to OPV at 3 months (p = 0.006) and 4 months of age (p = 0.008). This difference was most evident for the BF versus the MFGM group at 3 (p<0.001, effect size 0.56) and 4 months of age (p = 0.003, effect size 0.49). The corresponding numbers for the BF versus the SF group were p = 0.033, 0.37 and p = 0.039, 0.39, respectively. The pairwise comparison between the BF and F19 groups fell short of statistical significance at 3 months (p = 0.126) and 4 months of age (p = 0.06). There were no overall differences in the serum anti-diphtheria IgG concentrations among the groups (p>0.05), why no further pairwise comparisons were done.

**Table 2:** Concentration of specific IgG antibody responses to oral poliovirus (OPV) and to diphtheria vaccines in serum before and 4 weeks after the third dose of OPV (i.e. at 3 and 4 months of age) and the second dose of diphtheria (i.e at 4 and 5 months of age), respectively.

Immune marker	BF	SF	MFGM	F19	All groups compared	SF vs. MFGM	SF vs. F19	BF vs. SF	BF vs. MFGM	BF vs. F19
					*p-value*	*p-value*	*p-value*	*p-value*	*p-value*	*p-value*
Anti-OPV* IgG at 3 months	19.4** (15.8–23.9) n = 86	26.4 (21.5–32.3) n = 80	33.7 (28.2–39.9) n = 69	26.8 (21.6–33.5) n = 74	0.001	0.575	0.999	0.033	<0.001	0.126
Anti-OPV IgG at 4 months	31.9 (26.1–37.6) n = 85	38.4 (31.5–45.2) n = 78	45.0 (38.1–53.4) n = 71	37.8 (30.9–45.0) n = 75	0.005	0.696	0.999	0.039	0.003	0.06
Anti-diphtheria IgG at 4 months	0.05 (0.038–0.062) n = 65	0.05 (0.044–0.065) n = 69	0.06 (0.048–0.084) n = 66	0.06 (0.044–0.072) n = 57	0.107	NA	NA	NA	NA	NA
Anti- diphtheria IgG at 5 months	0.28 (0.221–0.331) n = 66	0.30 (0.237–0.369) n = 69	0.25 (0.185–0.322) n = 65	0.30 (0.223–0.373) n = 56	0.787	NA	NA	NA	NA	NA

BF; breast-fed reference group, SF; standard formula, MFGM; formula supplemented with milk fat globule membrane, F19; formula supplemented with *L*. *paracasei* ssp. *paracasei* strain F19.

*U/ml

** Geometric mean (95% Confidence Interval); NA; not assessed since the comparison of all groups were no statistically significantly different.

## Discussion

In support of our hypothesis, our findings demonstrate that feeding MFGM resulted in lower production of IL-2 and IL-17A compared with SF, and that the overall cytokine profile of the MFGM group approached that of the BF group. In contrast, and contrary to our hypothesis, feeding probiotic F19 resulted in slightly higher IL-2 while reducing IFN-γ production compared with SF. Consequently, both F19 and MFGM have immunomodulatory capacity. In comparison with SF, there was no immunomodulatory effect of F19 nor MFGM on the serum IgG antibody response to diphtheria or OPV vaccines thereby giving further support for their safe use in infancy.

IL-2 is a general T cell activating cytokine that induces effector and regulatory cells [27]. Here, we observed lower IL-2 concentrations in the MFGM group compared with the SF group. This finding is in agreement with a previous study that randomized Peruvian infants to intake of complementary foods with or without MFGM-supplementation during the second half of infancy [7]. In that study, there were lower IL-2 levels in the MFGM-supplemented group [28]. We also observed lower IL-17A concentrations in the MFGM group compared with SF. Increased levels of IL-17A have been reported in autoimmune diseases; however, IL-17A also plays a role in neonatal host defense and immunity, particularly in the defense against extracellular pathogens and fungal microbes [29]. As previously reported, there were no differences in growth nor any significant differences in the frequency of infections, episodes of fever, days with fever, or adverse events between the SF and MFGM groups [10] thereby demonstrating that the observed differences in IL-17A concentrations did not increase infection risk. Of note, all cytokine concentrations were comparable between the MFGM and the BF reference group and there were no differences among the formula groups in the concentrations of the other marker cytokines of infection or inflammation analyzed in the present study. The observed cytokine pattern of the MFGM group that approached that of the BF group may be associated with the previously reported finding [10] that all infectious outcomes for the MFGM group were close to those of the BF reference group, as opposed to the SF group that had more fever episodes and days with fever compared to BF.

In our previous study that randomized Swedish infants to intake of infant cereals with or without the addition of probiotic F19 during weaning [18, 20], F19 enhanced the IgG antibody response to diphtheria vaccine and induced Th1 and Th17 type responses. These findings were not replicated in the present study. Instead, we found increased IL-2 and reduced IFN-γ concentrations in the F19 compared with the SF group. These studies [18, 20] and the present study are not directly comparable as the vehicle of probiotic F19 (infant cereals versus infant formula), and also the dose, duration and timing of probiotic administration vary between these two studies. It is also likely that the probiotic response is modulated by the host, early environmental factors including delivery mode and infant diet, and the resident microbiota in the gastrointestinal tract [17]. We have not undertaken a comparison of gut microbiota between the Swedish [18, 20] and Chinese infants included in our studies, but a recent study found striking individual differences in the response to probiotic administration in adults, with variable changes of the gut microbiota across individuals [30]. It is also known that gut microbiota composition of infants varies across countries; Kuang et al reported the gut microbiota of Chinese infants to be abundant in Proteobacteria whereas that of Swedish infants was more variable [31]. Probiotics are theorized to confer their immunological effects either by modulation of the composition and functions of the resident microbiota in the gastrointestinal tract or by a direct influence on the immune system [17, 32]. Collectively, our results add to the growing notion that even the same probiotic strain may produce different results across individuals, and across populations.

Specific antibody responses function as a valuable proxy of immune ontogeny as they depend on the complex interaction between antigen presenting cells, specific T helper cells, intercellular cytokine signaling, and the differentiation of B cells to antibody producing plasma cells. Here, we took the opportunity to evaluate the booster response to a protein antigen (diphtheria toxoid) and a viral antigen (poliovirus), i.e. before and after the second dose of diphtheria and third dose of OPV, respectively. Compared to infants fed standard formula, a previous study [33] reported a lower IgG antibody response to poliovirus type 1 in French infants that received the same MFGM fraction as in the present study but as we found no differences among the formula groups, this indicates adequate immune ontogeny in all intervention groups and gives further support for the safe use of MFGM in infant formula. Differences in vaccine responses between breastfed and formula-fed infants have been repeatedly reported [9, 18, 34] and in the present study all formula groups had higher serum IgG antibody concentrations to OPV compared with the BF group. This difference was most evident for the MFGM group. Clinical studies have demonstrated that MFGM has a protective effect against infections [7, 9] and this is likely mediated by modulation of mucosal and systemic immune responses and the microbiota of the gastrointestinal tract [35]. The mechanisms behind these immunological effects are not yet fully elucidated, but glycoproteins and glycolipids present in MFGM are theorized to induce antimicrobial and anti-inflammatory functions in the gastrointestinal tract [36]. It also remains to characterize how both human MFGM and bovine MFGM concentrate are digested and where in the intestine bioactive proteins of MFGM are released from the MFGM complex [1].

We found no differences in the prevalence of doctor-diagnosed eczema at age 12 months among the groups, nor were there any differences in eczema severity as assessed by SCORAD. One previous study [33] reported a higher frequency of parent-reported eczema in infants fed MFGM while another study reported no increased risk of parent-reported rash [37]. Very recently, Li et al reported similar frequencies of rash and eczema in infants fed MFGM plus lactoferrin, compared with SF [38]. Collectively, our results do not indicate increased eczema risk in infants fed MFGM. We previously reported an eczema preventive effect by F19 [19]; however, the present study was not designed for eczema prevention. As the prevalence of eczema at 12 months was very low in all groups, a much larger sample size had been needed to examine eczema preventative effects.

This study has several strengths including the randomized controlled design and the large study population. Randomization was successful as there were no differences in demographic characteristics among the formula groups. In the non-randomized BF reference group, however, parental education level was higher, family size was smaller and gestational age was slightly longer compared with the combined formula groups. There is strong evidence from meta-analyses and systematic reviews that maternal education is an important factor for successful breastfeeding initiation and continuation [39–41]. This pattern was also seen is this multi-center study in China and is consistent with a recent Chinese cross-sectional study [42].

There are also limitations; these include the number of study sites, regional differences in immunization programs and the lack of a blood sampling at baseline for comparison of cytokine responses before and after the intervention period. An additional blood sampling after the IgG antibody peak response would have been needed to evaluate the memory response following immunization and should be considered in future studies, as should studies on the cellular immune response.

In summary, feeding probiotic F19 resulted in higher IL-2 while reducing IFN-γ production compared with SF whereas the cytokine profile of the MFGM group approached that of the BF group. No differences in vaccine responses to diphtheria or OPV were observed among the formula groups giving further support for their safe use in infants. The observed effects on the cytokine profile are likely induced either by direct influence of MFGM and F19 on the developing infant immune system, and/or by modulation of microbial composition and functions. Glycoproteins and glycolipids in MFGM are theorized to have both immunomodulatory and prebiotic effects [1]. Probiotics have potential to interact with the resident gut microbiota and to provide microbial stimuli for appropriate maturation of innate and adaptive immunity that protect from early infection [13, 17]. Future studies are warranted to examine the mechanistic effects of MFGM and specific probiotics on infant immune ontogeny and their impact on clinical outcomes.

## Supporting information

S1 Checklist CONSORT 2010 checklist of information to include when reporting a randomised trial*. (DOC)

S1 File Trial protocol. (DOC)
