# Supplementary material for: Serum cytokine patterns are modulated in infants fed formula with probiotics or milk fat globule membranes: A randomized controlled trial
Source: PLoS One. 2021 May 13;16(5):e0251293. doi: 10.1371/journal.pone.0251293 (PMC8118299; doi:10.1371/journal.pone.0251293)
Supplement: S1 File — (DOC) [file pone.0251293.s002.doc]

# Study Protocol Version 20120323 rev 20130827, REV 20141008

**Feeding infant formula with added probiotics or whey protein concentrate**

A double blind, randomized, controlled trial.

CONFIDENTIAL

This protocol was developed jointly by Sponsor, Principle Investigator and Co-principle Investigators.

RESPONSIBLE PERSONNEL

| Principal Investigator | Professor Bo Lonnerdal, Ph.D., Department of Nutrition, University of California Davis, USA.  Email: bllonnerdal@ucdavis.edu | ………..........……….………..  Date  .............................................  Signature |
| --- | --- | --- |
| Co-principle Investigator | Professor Olle Hernell, M.D., Ph.D., Department of Clinical Sciences, Pediatrics, Umeå University, Sweden.  Email: Olle.Hernell@pediatri.umu.se | ………..........……….………..  Date  .............................................  Signature |
| Co-principle  Investigator | Professor Yongmei Peng, M.D., Ph.D., Department of Child Health Care, Children's Hospital, Fudan University, Shanghai, China. Email: ympeng99@yahoo.com.cn | ………..........……….………..  Date  .............................................  Signature |
| Co-principle  Investigator | Professor Xiaonan Li, M.D., Ph.D., Children Health Care Department, Nanjing Medical University Affiliated Nanjing Children's Hospital, Nanjing, China.  Email: xiaonan6189@yahoo.com | ………..........……….………..  Date  .............................................  Signature |
| Sponsor | R&D Manager Preben Bødstrup Rasmussen  Arla Foods, Sønderhøj 14, 8260 Viby J, Denmark  Phone: +45 8938 1262,  Email: Preben.bodstrup.rasmussen@arlafoods.com | ………..........……….………..  Date  .............................................  Signature |

| Title page | 1 |
| --- | --- |
| Responsible personnel | 2 |
| Summary | 4 |
| Introduction | 7 |
| Objectives of trial | 7 |
| Hypotheses | 7 |
| Trial design | 8 |
| Study population | 8 |
| Product description | 9 |
| Outcomes | 9 |
| Conduct of trial | 12 |
| Description of visits | 13 |
| Statistical aspects and considerations | 14 |
| Ethical aspects | 15 |
| References | 16 |
| Appendices | 17 |

# SUMMARY/SYNOPSIS

| Feeding infant formula with added probiotics or whey protein concentrate |
| --- |
| Main researchers:  Professor Bo Lonnerdal, Professor Olle Hernell, Professor Yongmei Peng and Professor Xiaonan Li |
| Research centers:  Department of Child Health Care, Children's Hospital, Fudan University, Shanghai, China and Children Health Care Department, Nanjing Medical University Affiliated Nanjing Children's Hospital, Nanjing, China. |
| Aim:  To evaluate the effects of feeding an infant formula containing *L. paracasei ssp. paracasei* strain F19 or the Whey Protein Concentrate (Lacprodan MFGM) on episodes of infections, infant growth and health. |
| Primary outcome:  Infections episodes (gastrointestinal and acute respiratory infections diagnosed based on clinical signs and symptoms by study physician (number of episodes and duration))  Secondary outcomes:  Physician-diagnosed wheezing episodes (number of episodes and duration)  Physician-diagnosed/parent-reported eczema (number of episodes and duration)  Use of antibiotics (number of episodes and duration)  Unscheduled doctor’s visits and hospitalization (number of episodes and duration)  Stool consistency and frequency  Vomiting  Growth (body weight, length and head circumference)  Adverse events  Vaccine response after polio and diphtheria vaccination |
| Methods:  Double blind, randomized, controlled trial |
| Samples:  200 subjects in each of the three formula-fed groups and 200 subjects in the breast-fed reference group. In total, 800 infants will be included. |
| Inclusion criteria (formula-fed group):  healthy infants of mothers who could not or voluntarily resigned completely from breast-feeding at infant age 21 ± 7 days  Inclusion criteria (breast-fed group):  healthy infants exclusively breast-fed from birth and mothers intending to breastfeed >80% until at least 5^th^ month of age (meaning through the 4^th^ month of age).  Inclusion criteria (all infants):  delivered between 37 and 42 weeks of gestation  birth weight >2500 g and <4000 g  parent or the subject’s legal representative speak and understand Chinese  Exclusion criteria (formula-fed group):  fully or partially breast-fed infants  Exclusion criteria (breast-fed group):  infants fed >20% infant formula  Exclusion criteria (all infants):  malformations, handicaps or congenital diseases that could affect normal feeding or growth  treatment with antibiotics  fed infant formula with pre- and/or probiotics |
| Intervention and comparison:  Reference group: Breast-fed infants (>80% breastfed).  Arm 1: Infants fed standard infant formula  Arm 2 : Infants fed standard infant formula with addition of 10^9^ CFU of *L. paracasei* ssp. *paracasei* strain F19 per L ready-to-use infant formula.  Arm 3: Infants fed standard infant formula with the Whey Protein Concentrate (Lacprodan MFGM) 5 g per L ready-to-use infant formula.  Arm 1-3: The infants are fed standard infant formula until intervention, if infant formula feeding is started prior to inclusion.  Quantity, doses, administration route, duration time:  Infants will be orally fed with infant formula in quantities adequate for their weight, age and appetite.  Duration of intervention:  From 21 ± 7 days to 5^th^ month of age. Standard infant formula will be provided from 5^th^ to 7^th^ month of age (meaning from the beginning of the 5^th^ month of age through the 6^th^ month of age) in arm 1 - 3. Follow up until 13^th^ month of age (meaning through the 12^th^ month of age). |

Schematic overview of study:

| Visit (V) | V0 | V1 | V2 | V3 | V4 | V5 | V6 |  |  | V7 |  |  | V8 |
| --- | --- | --- | --- | --- | --- | --- | --- | --- | --- | --- | --- | --- | --- |
| Age  week (w)  months (m) | 3w | 1m | 2m | 3m | 4m | 5m | 6m | 7m | 8m | 9m | 10m | 11m | 12m |
| Enrollment | x |  |  |  |  |  |  |  |  |  |  |  |  |
| Randomization | x |  |  |  |  |  |  |  |  |  |  |  |  |
| Dispense study infant formula | x | x | x | x |  |  |  |  |  |  |  |  |  |
| Anthropometry  Body weight  Body length  Head circumference |  | x | x | x | X | x | x |  |  | x |  |  | x |
| Hand out of stool containers |  |  |  | x |  |  |  |  |  | x |  |  |  |
| Collection of stool samples | x* |  |  |  | X |  |  |  |  |  |  |  | x |
| Collection of blood samples |  |  |  | x | X |  |  |  |  |  |  |  |  |
| Review 3-day “Dietary and health journal” |  | x | x | x | X | X |  |  |  |  |  |  |  |
| Monitor primary endpoint |  | x | x | x | X | X | x | x | X | x | x | x | x |
| Review of secondary end-points and adverse events every two weeks |  | x | x | x | X | X | x | x | X | x | x | x | x |
| Intervention | xxxxxxxxxxxxxxxxxxxxxxxx | | | | |  | | |  |  |  |  |  |

‘* The stool sample from the breastfed infants may be collected at home at 21 ± 7 day of infant age and brought to the hospital at V1.

# INTRODUCTION

Breast-feeding is the ‘gold standard’ in early infant nutrition. However, for various reasons some mothers cannot or choose not to breast-feed. Therefore, one of the goals of infant formula development is to approach the composition and functionality of breast milk to give formula-fed infants as small a disadvantage as possible compared to breast-fed infants.

Differences between breast-fed and formula-fed infants still exist. Formula-fed infants have a greater tendency to develop gastrointestinal and respiratory infections as well as constipation and eczema (Agostoni *et al.* 2009, Van Rossum *et al.* 2008, EFSA, Scientific opinion 2009).

Some milk proteins have been demonstrated to have antimicrobial activities and can prevent diarrhea in small children. Arla Foods Ingredients has produced a bovine Whey Protein Concentrate (WPC) (Lacprodan MFGM) enriched in several bovine antimicrobial proteins and bioactive components. These components include the milk fat globule membrane (MFGM) proteins mucin 1, lactadherin and butyophilin as well as lactoferrin and sphingolipids. Several of the components in the WPC (Lacprodan MFGM) have been shown to have an inhibitory effect on bacterial and viral pathogens, particularly rotavirus, whereas other components were shown to facilitate nutrient utilization by intestinal cells (Petterson *et al.* 2001, Tian *et al.* 2010). A randomized double blind controlled trial in children aged 6 to 11 months on the WPC (Lacprodan MFGM) at a daily dose of approx. 8.22 g showed a reduced overall diarrhea incidence and prevalence (Zavaleta *et al.* 2011). Therefore, it is likely that the addition of the WPC (Lacprodan MFGM) to the diet of infants reduce the incidence of diarrhea. The test diet was well accepted by the infants and was the main source of micronutrient intake (Zavaleta *et al.* 2011).

Breast-fed infants have been shown to have a gut microbiota more dominated by bifidobacteria and lactobacilli compared to formula-fed infants. In contrast, the microbiota of formula-fed infants is more diverse, containing Bacteroides, Bifidobacteria, Staphylococci, Escherichia coli and Clostridia (Harmsen *et al.* 2000). These observed differences have been suggested to contribute to the lower incidence of infections, allergies and gastrointestinal disturbances in breast-fed compared with formula-fed infants (Koletzko *et al.* 1998, Agostoni *et al.* 2009). If this is the case, it seems reasonable to develop infant formulas to support the establishment of a microbiota, which resembles that of breast-fed infants, for example by adding probiotics. A previous study indicated that feeding *Lactobacillus paracasei* ssp. *paracasei* strain F19 during weaning could be an effective tool in the prevention of early manifestation of allergy, e.g., eczema in a group of infants aged 4 to 13 months (West *et al.* 2009).

The importance of testing new ingredients for use in infant formula is stressed in the European Directive “COMMISSION DIRECTIVE 2006/141/EC of 22 December 2006 on infant formulae and follow-on formulae, amending Directive 1999/21/EC. It reads: ”*It is important that ingredients used in the manufacture of infant formulae and follow-on formulae are suitable for the particular nutritional use by infants and that their suitability has been demonstrated, when necessary, by appropriate studies”*

The study mentioned above in a group of infants aged 4 to 13 months fed *L. paracasei* ssp. *paracasei* strain F19 during weaning showed no negative effect on either tolerance or growth (West *et al.* 2008, West *et al.* 2009, http://umu.diva-portal.org/smash/record.jsf?pid=diva2:141793). *L. paracasei* ssp. *paracasei* strain F19 is approved for use in infant formula by the Ministry of Food, Agriculture and Fisheries, Danish Veterinary and Food Administration (Appendix 1).

Likewise as mentioned previously the WPC (Lacprodan MFGM) was tested in complementary foods in a group of infants aged 6 to 11 months showing adequate growth (Zavaleta *et al.* 2011).

# OBJECTIVES OF THE TRIAL

## Main objective

The purpose of the present study is to evaluate the effect of feeding infants *L. paracasei* ssp. *paracasei* strain F19 or a WPC (Lacprodan MFGM) in a standard infant formula on episodes of infections.

Infants will receive a standard infant formula from the time the mother starts abandoning breast-feeding. If the infant is fully formula-fed at 21 ± 7 days of age, he or she will be randomized to one of the three study groups and receive the assigned infant formula until the 5^th^ month of age (meaning through the 4^th^ month of age). From the 5^th^ to the 7^th^ month of age (meaning from the beginning of the 5^th^ month of age through the 6^th^ month of age), all infants in the three formula arms will receive standard formula.

From inclusion until the 13^th^ month of age (meaning through the 12^th^ month of age), the incidence and duration of infections (gastrointestinal, acute respiratory tract infections), eczema (V8) and wheezing is recorded based on diagnosis by study physician. Furthermore, parent-reported eczema, the number of days with fever (>38 °C), vomiting, use of antibiotics, unscheduled doctor’s visits and hospitalization will be registered based on reviews performed every second week by the study physician/nurse. Stooling habits will be reported in “Dietary and health questionnaire”. Growth and adverse events will be followed by the study physician/nurse.

Vaccine response after polio and diphtheria vaccination (part of the polio and diphtheria, pertussis and tetanus (DPT) vaccine) will be measured after the second DPT and third polio vaccination.

# Hypothesis

Addition of *L. paracasei* ssp. *paracasei* strain F19 or a WPC (Lacprodan MFGM) will reduce infections in formula-fed infants, improve their health and make them more similar to breast-fed infants.

# TRIAL DESIGN

## Type of trial

The study is a randomized, double-blind, controlled study in which infants whose mothers could not or voluntarly decided not to breastfeed are randomized to receive a standard infant formula, or the same standard formula supplemented with *L. paracasei* ssp. *paracasei* strain F19 in a dose of 10^9^ CFU per L ready-to-use infant formula or a WPC (Lacprodan MFGM) 5 g per L ready-to-use infant formula from 21 ± 7 days to the 5^th^ month of age. Exclusively breast-fed infants are included as a reference group.

## Subjects, groups and centers

The study will be conducted at Nanjing Childrens Hospital, Nanjing Medical University, Nanjing and Childrens Hospital, Fudan University, Shanghai. The target population is healthy, term infants of mothers who could not or voluntarily decided not to breast-feed their infants.

## Sample size

A sample size of 540 (180 in each group) provides 80% power (5% significance) to detect a difference of 20% in incidence of infectious episodes. Anticipating a drop-out rate of between 15 and 20%, 200 infants will be included in each of the study groups (in total 600 infants). A breast-fed group will be included as reference (200 infants). In total, 800 infants will be included.

# STUDY POPULATION

## Description

Healthy, term infants of mothers who cannot or voluntarily decide not to breast-feed will be eligible to participate in the study and will be randomized to one of the following treatment groups:

Arm 1: standard infant formula.

Arm 2: standard infant formula with addition of *L. paracasei*, ssp. *paracasei* strain F19 at a dose of 10^9^ CFU per L ready-to-use infant formula.

Arm 3: standard infant formula in which 27.5 g of the total 113 g protein per kg infant formula powder (24%) is substituted with a WPC (Lacprodan MFGM) resulting in a dose of 5 g per L ready-to-use infant formula.

The formula fed infants will be recruited and randomized at 21 ± 7 days of life. Prior to start of intervention they will be fed standard infant formula if formula feeding has been started. The breast-fed reference group will be recruited at the same time, but will not be randomized. All infants in this group have to be fully breast-fed from birth and the mother’s intention should be to continue breast-feeding >80 % to the 5^th^ month of age. If breastmilk supply is insufficient, the breastfed infants will be supplemented with standard infant formula <20%.

## Subject inclusion criteria

Subjects recruited must comply with all the following inclusion criteria:

**Formula-fed group:**

-healthy infants of mothers who cannot or voluntarily resign completely from breast-feeding

at infant age 21 ± 7 days.

**Breast-fed group:**

- healthy infants exclusively breast-fed from birth and with the mother’s intention to breast-feed > 80% until at least to the 5^th^ month of age.

**All infants:**

- delivered between 37 and 42 weeks of gestation

- birth weight >2500 g and <4000 g

- parent or the subject’s legal representative speak and understand Chinese

## Subject exclusion criteria

Subjects presenting one or more of the following criteria are excluded from participation:

**Formula-fed group:**

- fully or partially breast-fed infants at 21 ± 7 days of age

**Breast-fed group:**

- infants fed infant formula >20% infant formula

**All infants:**

- malformations, handicaps or congenital diseases that could affect normal feeding or growth

- treatment with antibiotics

- fed infant formula with probiotics

# Product description

Control infant formula (all formula-fed infants prior to start of intervention and from the 5^th^ to the 7^th^ month of infant age, arm 1 during intervention, and breastfed infants up to 20% if needed): A standard infant formula, which fulfills the Chinese legislation regarding composition of infant formula for use in infants from 0 to 6 months of age. For specific infant formula composition, see Appendix 2 Table 1.

Study infant formula 1 (arm 2): Standard infant formula with addition of 10^9^ CFU of the probiotic bacterium *L. paracasei* ssp. *paracasei* strain F19 per L ready-to-use infant formula, see Appendix 2 Table 2. The safety aspects regarding the probiotic bacterium *L. paracasei*, ssp. *paracasei* strain F19 are summarized in Appendix 1 and 3.

Study infant formula 2 (arm 3): Standard infant formula where 27.5 g of total protein per kg is substituted with protein from WPC (Lacprodan MFGM) resulting in a concentration of WPC (Lacprodan MFGM) of 5 g per L ready-to-use infant formula, see Appendix 2 Table 3. The safety aspects regarding the WPC (Lacprodan MFGM) are summarized in Appendix 4.

The infant formulas used in the present study are manufactured from imported milk powder (from Arla Foods, Denmark), WPC (Lacprodan MFGM) (from Arla Foods Ingredients, Denmark) and *L. paracasei*, ssp. *paracasei* strain F19 (from Chr. Hansen, Denmark), all produced in Denmark. The infant formulas are manufactured at Arla Foods` production site in Hohot, China in accordance with Danish and EU regulations under hygienic conditions and adhere to all prerequisites for human consumption according to the European Union legislation.

## Blinding technique

This study is blinded to the sponsor, study researchers and participants until the last follow-up and the statistical analysis have been performed. The blinding is performed at the infant formula production site within the company of the sponsor (Arla Foods). There will be no difference in smell and taste of the three infant formulas (the control and the two study infant formulas will only be differential to the sponsor, study researchers and participants by a color code – a separate color code will be used for the standard infant formula used before and after intervention and for the breastfed reference group in order to keep the blinding of the intervention groups). One person at the production site not directly connected to the study will keep the code until the statistical analysis has been performed.

## Treatment administration

Before intervention arm 1-3 will be fed standard infant formula ad libitum. During intervention arm 2 and 3 will receive the study infant formulas ad libitum. Arm 1 and the breast-fed group will also be fed standard infant formula and breastmilk (the breastfed group can be supplemented with <20% standard formula if breast milk supply is insufficient), respectively, ad libitum.

The infants (arm 1-3) will be fed the study or standard infant formulas exclusively from 21 ± 7 days of age until the 5^th^ month of age.

From the 5^th^ to the 7^th^ month of age, arm 1-3 will receive standard infant formula ad libitum.

Complementary foods will not be allowed during the intervention (until the 5^th^ month of infant age), but should be introduced no later than from 26 weeks of age according to current recommendations. The complementary food provided to the infants must not contain probiotics.

## Route of administration

Infants will receive the control or study infant formulas orally.

## Subject compliance

The parent or the subject’s legal representative will be asked to keep a „Dietary and Health journal” for three consecutive days each month up to the 5^th^ month of age for participating infants. This will allow the study staff to check compliance with infant formula consumption, and also to confirm that the infants are receiving volumes of infant formula appropriate for their nutritional and fluid requirements.

The parent or the subject’s legal representative of the breastfed infant will be asked to note all infant formula fed to the infant to allow the study staff to check compliance with breast milk consumption.

# Outcomes

## Clinical outcomes:

Primary outcome:

Incidence and duration of infectious episodes (acute diarrhea, acute respiratory tract infections and fever) diagnosed by the study physician based on the definitions of:

**Acute diarrhea** as three or more watery stools within a 24-hour period or loose-to-watery bowel movements that exceed the child’s usual daily stool frequency by two or more stools as our standard practice.

**Acute respiratory infections** will be defined according to the presence of 2 or more of the following symptoms as reported by the mother nasal discharge (clear, cloudy, yellow, or green), cough, fever, rapid, labored and/or noisy breathing, wheezing, chest indrawing, flaring of nostrils, ear pain and/or discharge, and cyanosis. Mild (the presence of nasal discharge only) or moderate to severe (the presence of 2 or more of the above symptoms). Respiratory symptoms that occur within 2 weeks of the beginning of the illness will be defined as part of the same episode. Symptoms presented more than 2 weeks after the start of an incident will be considered as a new episode. Such an instance will merit a new nasopharyngeal aspiration.

**Fever** >38 °C.

Secondary outcomes:

Wheezing episodes diagnosed by the study physician defined as a high-pitched whistling sound associated with obstructive breathing. The length of the period with wheezing will be recorded.

Eczema until 12 months of infant age will be parent-reported based on Atopic Dermatitis Quickscore (ADQ) (Carel *et al.* 2008) and at V8 (infant age 12 months) eczema will be diagnosed by the study physician based on the SCORAD system, see Appendix 5 (Anonymous 1993, http://adserver.sante.univ-nantes.fr/Compute.html). The length of the period with eczema will be recorded.

Specific IgG response after the first DPT and the second polio vaccination (w when the infant is 3 months of age (visit 3)), and 4 weeks after

Use of antibiotics (reviewed by the study physician). The length of the period with antibiotic use will be recorded.

Unscheduled doctor’s visits and hospitalization (incidence, duration, diagnosis and treatment is reviewed by the study physician). The length of the hospitalization period will be recorded.

Vomiting (reviewed by the study physician)

Adverse events (reviewed by the study physician)

Growth (body weight, length, head circumference measured by the study physician)

Stool consistency and frequency (parent reported). Stool consistency will be registered as watery diarrhea, loose, soft formed or hard in the 3-day “Dietary and health journal” (Appendix 8).

## Morbidity measurements and adverse events

When gastrointestinal, acute respiratory infections or wheezing is suspected, the study physician should be contacted for diagnosis.

Days with fever (>38 °C), vomiting, use of antibiotics, unscheduled doctor’s visits and hospitalization (diagnosis and treatment) and adverse events (Appendix 6 and 7) will be reviewed with the parent or the subject’s legal representative by the study physician/research nurse every two weeks during scheduled visits or a phone call.

Stool consistency and frequency will be registered by the parent or the subject’s legal representative for 3 consecutive days every month.

### Definition of adverse event

An adverse event (AE) is defined as any untoward occurrence in a subject, or clinical investigation subject administered an investigational product, and which does not necessarily have to have a causal relationship with this treatment.

AEs are illnesses, signs or symptoms (including an abnormal laboratory finding) occurring or worsening in the course of the study. AEs can be serious or minor. They may or may not lead to the withdrawal of the subject from the study.

Investigators must know and record the following information about AEs:

Date

Duration

Frequency

Intensity

Seriousness

Measures taken

Sequelae

Relationship to test products

Intensity of adverse event is described as:

Mild: symptoms hardly perceived, only slight impairment of general well being

Moderate: clearly noticeable symptom, but tolerable without immediate relief

Severe: overwhelming discomfort

### Definition of serious adverse event

A serious adverse event (SAE) is a fatal or life threatening event causing permanent harm or requiring/extending in-patient treatment at a hospital or which the physician considers medically relevant.

Non-serious: all other AEs not corresponding to the definition of a SAE, are considered non-serious. In the case of a SAE the infant will be taken off the study.

Relation to test product:

The study physician will assess the possibility of a link between the study infant formula and adverse event on the basis of the following criteria:

Unrelated: There is an obvious other explanation for the AE, e.g.:

The AE is obviously explained by the subject’s disease

The AE is in accordance with the effect or adverse effect of concomitant medication

The AE has occurred already prior to the administration of the study product

Unlikely relation: Reasonable temporal relationship with the intake of the study product, but there is another plausible explanation for the occurrence of the AE

Probable relation: Reasonable temporal relationship with the intake of the study product and plausible reasons point to a causal relationship with the study product

Certain relation: Reasonable temporal relationship with the intake of the study product and there is no other explanation for the AE and subsidence or disappearance of the AE on withdrawal of the study product (de-challenge) and recurrence of the symptoms on rechallenge

Cow´s milk protein allergy will be diagnosed by a physician (by de-challenge/challenge with same symptoms) and treated with a protein hydrolysate (drop out) but will remain in the study for ITT analysis

### Reporting and documentation of an adverse event

All adverse events must be documented on the appropriate pages of the case report form (see appendices 5 and 6).

### Follow up of an adverse event

In the case of a serious adverse event persisting beyond the trial termination, a follow up visit may be required. Further, in the event that additional analyses are required for the evaluation of a potential cause-effect relationship between the study products and the adverse event, all examinations and laboratory analyses and their results will be documented in the case report form or in an attached file.

## Anthropometry

The weight is taken to the nearest 10 g. Infants will be weighed naked, i.e. without clothing or nappy/diaper, on electronic weighing scales. The same scales will be used for all infants at all visits. The electronic weighing scales will be calibrated as per the manufacturer’s recommendations at V1 and every visit thereafter until the end of the study.

Recumbent length measured to nearest 1 mm. Infants will be measured using a standardized length board. At least two people will be present to maintain proper body alignment and full body extension with feet flexed. Head circumference will be measured to nearest 1 mm. Head circumference will be obtained using a standard non-elastic plastic coated measuring tape. The measurement will be taken approximately 2.5 cm above the eyebrows, directly over the largest circumference of the skull.

## Stool sample collection and storage

The parent or subject’s legal representative is asked to collect stool samples at infant age 14 ± 3 days of age, just prior to V4 and V8 (at 4 and 12 months of age, respectively). Before collection of the first stool sample, a reusable isolated bag for transportation of the stool samples, a reusable freezing body, two small containers for the stool samples, a plastic bag for the filled containers, gloves and instructions for collection, storage and transportation of the stool samples will be provided to the parent or the subject’s legal representative.

At V3 and V7, two containers for stool samples, a plastic bag for the filled containers, gloves and instructions for collection, storage and transportation of the stool samples will provided to the parent or the subject’s legal representative.

Prior to V4 and V8 the study staff will make a phone call to remind the parent or the subject’s legal representative to collect the stool samples.

The stool samples will be collected in the containers and put in the plastic bag prior to V4 and V8 and stored in the freezer (-20 °C) until the day of the visit. The frozen stool samples will be transported to the study site together with the frozen freezing body in the isolated bag.

At study sites, the samples will be stored at –20 °C until they are shipped on dry ice to the appropriate laboratory for analysis.

## Stool sample analysis

To measure compliance, the presence of *L. paracasei*, ssp. *paracasei* strain F19 will be determined by RT-PCR in the stool sample collected at V4 in collaboration with Ulla Svensson (Arla Foods Stockholm, Sweden). 100 randomized samples will be analyzed in each of the three study groups. If *L. paracasei*, ssp. *paracasei* strain F19 is identified in less than 80% of the stool samples from the group of infants treated with infant formula containing *L. paracasei*, ssp. *paracasei* strain F19, the remaining samples collected at V4 in this group will be analyzed.

The gut microbial composition will be analyzed in a subset of the stool samples by pyrosequencing technology in collaboration with SMI, Stockholm, Sweden or UCDavis, USA.

## Blood sample collection and storage

When the infant is 3 months of age (at visit 3) of age just prior to the first DPT and second polio vaccination and 4 weeks after, blood samples will be collected by either venous blood sampling or finger prick by the study staff. 30-60 min. prior to sample collection, EMLA cream will be applied to the infant’s finger or arm as local anesthetic. A minimum of 0.5 ml and a maximum 2 ml of blood will be collected at each collection.

Serum samples will be stored at –80 °C until they are shipped on dry ice to the appropriate sites for analysis.

## Blood sample analysis

The serum samples will be analyzed for antibodies against dipteria toxin and poliovirus (laboratory for analysis to be decided). Amino acids, blood urea nitrogen, ferritin (Professor Bo Lönnerdal, UCDavis, USA), cytokines, adiponectin, leptin and ghrelin (Professor Olle Hernell, Umeå University) will be analyzed prioritized as mentioned (prioritization could be subjected to changes depending on new information gained during the study).

# Conduct of the trial

## Study plan

## Please refer to the study plan chart at page 6.

## Subject recruitment

Subjects will be recruited from the following centers: Children’s Hospital, Nanjing and Children's Hospital, Fudan University, Shanghai. The expected inclusion rate in the study is 15 infants per month at each center (In total, 30 infants per month for the two study sites).

The mothers and the newborn infants will spend approximately the first week after birth in hospital. During this week, the study staff will screen the infants for eligibility. The study staff will explain about the study and ask how breastfeeding is progressing.

If the mother intends to breast-feed her infant and the infant meets all inclusion criteria and does not present any of the exclusion critera for the breastfed group, the mother will be asked whether she is interested in her infant’s participation in the breast-fed reference group in the study.

- Furthermore, the study staff will ask the mother if she accepts the study staff to call her when her infant is 2 weeks of age to ask the status of feeding. If she agrees, she will be called again at 2 weeks of infant age. If informed concent is obtained, the infant will be enrolled in the study at 3 ± 1 weeks of age.

If the mother has decided to feed her infant infant formula, if the infant meets all inclusion criteria and does not present any of the exclusion criteria for the formula fed groups, the mother will be asked whether she is interested in her infant’s participation in the study. If yes, the study staff will ask the mother if she accepts that the study staff to call her at 2 weeks of infant age to ask the status of feeding. If she agrees, the study staff will provide her the standard infant formula (enough for 2 weeks) and call her at 2 weeks of infant age. She will be called again at 2 weeks of infant age and asked how the status of feeing is. If she at the call at 2 weeks after birth is breast feeding she is asked if she accepts the study staff to call her again after 1-2 more weeks. If the infant is fully formula fed and informed concent is obtained, the infant will be enrolled in the study at 3 ± 1 weeks of age.

## Economic relationship

The parents or the subject’s legal representatives of the infants do not receive any fee for participation in the study. The infant formulas used before (until infant age 21 ± 7 days), during the time of intervention (until the 5^th^ month of infant age in the three arms of the study and as supplement in the breastfed reference group if breast milk supply is insufficient) and after intervention (from the 5^th^ to the 7^th^ month of infant age) will be sponsored by Arla Foods and free of charge for the parents or the subject’s legal representatives. Coupons for diapers or puré (from the 5^th^ month of infant age) will be provided to the parents or the subject’s legal representatives in the breast-fed reference group (purées on parental request, and altogether with a total value similar to that of the infant formula provided to the formula-fed infants). Traffic cards will be provided to the parents to cover transportation expences related to the study. A small fairytale book will be given to the parents or the subject’s legal representatives for participation in the study.

## Researcher qualifications

Researchers participating in the study are medical doctors, pediatricians, and specially educated study nurses. All staff is qualified to perform all procedures required for the conduct of the study (collecting blood samples, examining the infant, diagnosing diarrhea and acute respiratory infections, taking anthropometric measurements, etc).

## Study center equipment

Both centers are fully equipped with all needed facilities and utensils for conducting the study such as: examination rooms, infant scales, length boards and freezers for preserving stool and blood samples.

## Timeline for study

# DESCRIPTION OF THE VISITS

## Before baseline visit

A reusable isolated bag for transportation of the stool samples, reusable freezing bodies, two small containers for the stool samples, a plastic bag for the filled containers, gloves, and instructions for collection, storage and transportation of the stool samples will be provided to the parent or the subject’s legal representative before the infant is 21 ± 7 days of age.

## V0 (baseline visit at 21 ± 7 days of age for formulafed infants)

The following data will be collected at time of enrolment:

the parents’ or the legal representatives’ name, address and phone number, demographic data of the subject: date of birth, birth order (older siblings), pregnancy complications, mode of delivery, delivery complications, gestational age, sex, birth weight, length and head circumference, APGAR score at 1, 5 and 10 minutes, hereditary diseases in the family (including eczema in siblings and parents), the infant´s medical history, previous or current medical treatments or supplements and probiotics intake during pregnancy.

The following steps will be performed at baseline visit:

enrol subject

randomize to one of the assigned groups (assign to breast-fed infants to breast-fed group)

dispense of the study infant formula

collect stool samples

dispense 3-day “Dietary and health journal”

explain how to complete the 3-day “Dietary and health journal”

schedule next visit

## V1 (1 month of age)

perform anthropometric measurements

collect and review the records in the 3-day “Dietary and health journal”

review days with fever (>38 °C), vomiting, parent-reported eczema based on ADQ, use of antibiotics, unscheduled doctor’s visits/hospitalization (diagnosis, duration and treatment) and adverse events/concomitant medication with the parent or the subject’s legal representative

dispense study infant formula

collect stool samples (from breast-fed infants)

dispense 3-day “Dietary and health journal”

schedule next visit

## V2 (2 months of age)

perform anthropometric measurements

collect and review the records in the 3-day “Dietary and health journal”

review days with fever (>38 °C), vomiting, parent-reported eczema based on ADQ, use of antibiotics, unscheduled doctor’s visits/hospitalization (diagnosis, duration and treatment) and adverse events/concomitant medication with the parent or the subject’s legal representative

dispense study infant formula

dispense 3-day “Dietary and health journal”

schedule next visit

## V3 (3 months of age)

perform anthropometric measurements

collect and review the records in the 3-day “Dietary and health journal”

review days with fever (>38 °C), vomiting, parent-reported eczema based on ADQ, use of antibiotics, unscheduled doctor’s visits/hospitalization (diagnosis, duration and treatment) and adverse events/concomitant medication with the parent or the subject’s legal representative

collect blood sample (prior to vaccination)

give DPT and polio vaccination (as part of the vaccination programme)

dispense study infant formula

provide containers for stool samples, a plastic bag and a description of collection, storage and transportation of stool samples

dispense 3-day “Dietary and health journal”

schedule next visit

**Before V4 and V8.**

Prior to V4 and V8 the investigators will remind the parent or the subject’s legal representative to collect stool samples.

## V4 (4 months of age (4 weeks after V3))

perform anthropometric measurements

collect and review the records in the 3-day “Dietary and health journal”

review days with fever (>38 °C), vomiting, parent-reported eczema based on ADQ, use of antibiotics, unscheduled doctor’s visits/hospitalization (diagnosis, duration and treatment) and adverse events/concomitant medication with the parent or the subject’s legal representative

collect stool samples

collect blood sample

dispense standard infant formula

dispense 3-day “Dietary and health journal”

schedule next visit

## V5 (5 months of age)

perform anthropometric measurements

collect and review the records in the 3-day “Dietary and health journal”

review days with fever (>38 °C), vomiting, parent-reported eczema based on ADQ, use of antibiotics, unscheduled doctor’s visits/hospitalization (diagnosis and treatment) and adverse events/concomitant medication with the parent or the subject’s legal representative

dispense standard infant formula

schedule next visit

## V6 (6 months of age)

perform anthropometric measurements

review days with fever (>38 °C), vomiting, parent-reported eczema based on ADQ, use of antibiotics, unscheduled doctor’s visits/hospitalization (diagnosis, duration and treatment) and adverse events/concomitant medication with the parent or the subject’s legal representative

dispense control infant formula

schedule next visit

## V7 (9 months of age)

perform anthropometric measurements

review days with fever (>38 °C), vomiting, parent-reported eczema based on ADQ, use of antibiotics, unscheduled doctor’s visits/hospitalization (diagnosis, duration and treatment) and adverse events/concomitant medication with the parent or the subject’s legal representative

provide containers for stool samples, a plastic bag and a description of collection, storage and transportation of stool samples

schedule next visit

## V7 (12 months of age)

perform anthropometric measurements

perform an evaluation of the presence of eczema based on SCORAD

review days with fever (>38 °C), vomiting, parent-reported eczema based on ADQ, use of antibiotics, unscheduled doctor’s visits/hospitalization (diagnosis, duration and treatment) and adverse events/concomitant medication with the parent or the subject’s legal representative

collect stool samples

provide fairytale book

**In between visits**

Between the visits, the study staff will make a phone call to the parent or the subject’s legal representative to review days with fever (>38 °C), vomiting, parent-reported eczema based on ADQ, use of antibiotics, unscheduled doctor’s visits and hospitalization (diagnosis, duration and treatment) and adverse events and concomitant medication with the parent or the subject’s legal representative.

# STATISTICAL ASPECTS AND CONSIDERATIONS

## Randomization

Infants who are eligible and with consenting parents or subject’s legal representatives will be randomized at the study sites using a computer program using permuted block randomization ensuring equal distribution of gender.

## Statistical analysis

All analysis will be based on the intention to treat principle. Non-normal distributed data will be log transformed to ensure normal distribution. For all parametric data Student’s t-test, One-way ANOVA and repeated measures ANOVA will be used. For statistically significant results we will consider those, in which p<0.05. The data will be analyzed using SPSS with guidance of a statistician.

## Intention to treat analysis

Data from all randomized subjects will be considered in the intention to treat analysis.

# Ethical aspects

## Breast feeding

## It is well acknowledged by the investigators that breast milk is the optimal food for the infant. This will be communicated to the mothers and only infants of mothers who could not or voluntarily resigned from breast-feeding will be taken into account for participation in the study. There will be no attempt to promote conversion from breast-feeding to formula feeding. Researchers will discuss the benefits of breast-feeding with mothers prior to inclusion to the study.

Arla Foods (The sponsor) is very well aware of the dilemma of marketing infant formula. The International Code of Marketing of Breast-milk Substitutes (The International Code of Marketing of Breast-milk Substitutes World Health Organization Geneva 2008) is very well known and fully met by all employees of Arla Foods developing, marketing and selling breast milk substitutes.

## Blood sampling

There may be a little discomfort for the infant associated with blood sampling. However, by applying the EMLA cream very little pain will be experienced by the infant. A trained nurse will do the blood sampling following established hygienic routines of the hospital, thus there is minimal risk of infection and a bruise at the place of collection.

## Informed consent

When the investigator has determined that the subject is an appropriate candidate for the study, the study will be described and explained orally to the subject’s parents or legal representatives. The investigator will answer all questions fully. A copy of the information sheet will be given to the subject’s parents or legal representatives (Appendix 9).

Written, informed consent will be obtained from the subject’s parents or legal representatives by the investigator prior to enrolment in the study (Appendix 9). The consent form will be signed and dated by both parents or legal representatives and the investigator. The consent form will be completed in two copies: the first copy is kept in the investigator’s file and the second is given to the parents or legal representatives. No subject will receive treatment before completion of the written informed consent.

## Ethics committee approval

The study protocol will be submitted for examination to the Institutional Ethics Committee by the principal investigator. Commencement of the clinical trial is not permitted without written approval of the Ethics Committee. The Ethics Committee will be notified of all subsequent additions or changes in the study protocol.

# REFERENCES

Agostoni C, Braegger C, Decsi T, Kolacek S, Koletzko B, Michaelsen KF, Mihatsch W, Moreno LA, Puntis J, Shamir R, Szajewska H, Turck D, van Goudoever J (2009) Breast-feeding: A Commentary by the ESPGHAN Committee on Nutrition Journal of Pediatric Gastroenterology and Nutrition 49:112–125

Anonymous (1993) Severity scoring of atopic dermatitis: the SCORAD index. Consensus Report of the European Task Force on Atopic Dermatitis. Dermatology 186:23–31

Carel K, Bratton DL, Miyazawa N, Gyorkos E, Kelsay K, Bender B, Strand M, Atkins D, Gelfand EW, Klinnert MD (2008) [The Atopic Dermatitis Quickscore (ADQ): validation of a new parent-administered atopic dermatitis scoring tool.](http://www.ncbi.nlm.nih.gov/pubmed/19055204) Ann Allergy Asthma Immunol 101:500-7

COMMISSION DIRECTIVE 2006/141/EC of 22 December 2006 on infant formulae and follow-on formulae and amending Directive 1999/21/EC

Harmsen HJ, Wildeboer-Veloo AC, Raangs GC, et al. (2000) Analysis of intestinal flora development in breast-fed and formula-fed infants by using molecular identification and detection methods. J Pediatr Gastroenterol Nutr 30, 61–67

<http://umu.diva-portal.org/smash/record.jsf?pid=diva2:141793>

Internatinal Code of Marketing of Breast-milk Substitutes World Health Organization Geneva 2008

Koletzko B, Aggett PJ, Bindels JG, et al. (1998) Growth, development and differentiation: a functional food science approach. Br J Nutr 80, 5–45

Peterson JA, Scallan CD, Ceriani RL, Hamosh M (2001) [Structural and functional aspects of three major glycoproteins of the human milk fat globule membrane.](http://www.ncbi.nlm.nih.gov/pubmed/11787681) Adv Exp Med Biol 501, 179-87

Pneumonia the forgotten killer of children, UNICEF and WHO

SCIENTIFIC OPINION (2009) Scientific Opinion on the appropriate age for introduction of complementary feeding of infants. EFSA Journal 7:1423

[Tian H](http://www.ncbi.nlm.nih.gov/pubmed?term="Tian H"%5BAuthor%5D), [Maddox IS](http://www.ncbi.nlm.nih.gov/pubmed?term="Maddox IS"%5BAuthor%5D), [Ferguson LR](http://www.ncbi.nlm.nih.gov/pubmed?term="Ferguson LR"%5BAuthor%5D), [Shu Q](http://www.ncbi.nlm.nih.gov/pubmed?term="Shu Q"%5BAuthor%5D) (2010) Influence of bovine lactoferrin on selected probiotic bacteria and intestinal pathogens. Biometals. 23, 593-6

Van Rossum CMT, Bu¨chner FL, Hoekstra J (2008) Quantification of health effects of breastfeeding. Review of the literature and model situation. RIVM Report 350040001/2005. Available at: http://www.rivm.nl/bibliotheek/rapporten/350040001.pdf. Accessed December 13, 2008.

West CE, Gothefors L, Granström M, Käyhty H, Hammarström ML, Hernell O (2008) Effects of feeding probiotics during weaning on infections and antibody responses to diphtheria, tetanus and Hib vaccines. Pediatr Allergy Immunol 19, 53-60

West CE, Hammarström ML, Hernell O. (2009) Probiotics during weaning reduce the incidence of eczema. Pediatr Allergy Immunol 20, 430-7

Zavelta N, Kvistgaard AS, Graverholt G, Respicio G, Guija H, Valencia N, Lönnerdal B (2011) Efficacy of a Complementary Food Enriched with a Milk Fat Globule Membrane Protein Fraction on Diarrhea, Anemia and Micronutrient Status in Infants. J Pediatr Gastroenterol Nutr. May Epub ahead of print.

# Appendices

| Appendix 1 | Ministry of Food, Agriculture and Fisheries  Danish Veterinary and Food Administration approval of *L. paracasei ssp paracasei* strain F19 for use in infant formula |
| --- | --- |
| Appendix 2 | Infant formula composition |
| Appendix 3 | Summary on safety aspects of *L. paracasei ssp paracasei* strain F19 |
| Appendix 4 | Summary on safety aspects of the WPC (Lacprodan MFGM) |
| Appendix 5 | SCORAD evaluation sheet |
| Appendix 6 | Adverse events form |
| Appendix 7 | Serious adverse events form |
| Appendix 8 | “Dietary and health journal” |
| Appendix 9 | Parents/subject’s legal representatives’ study information and Informed consent form |

APPENDIX 1. Ministry of Food, Agriculture and Fisheries, Danish Veterinary and Food Administration approval of *L. paracasei ssp paracasei* strain F19 for use in infant formula.


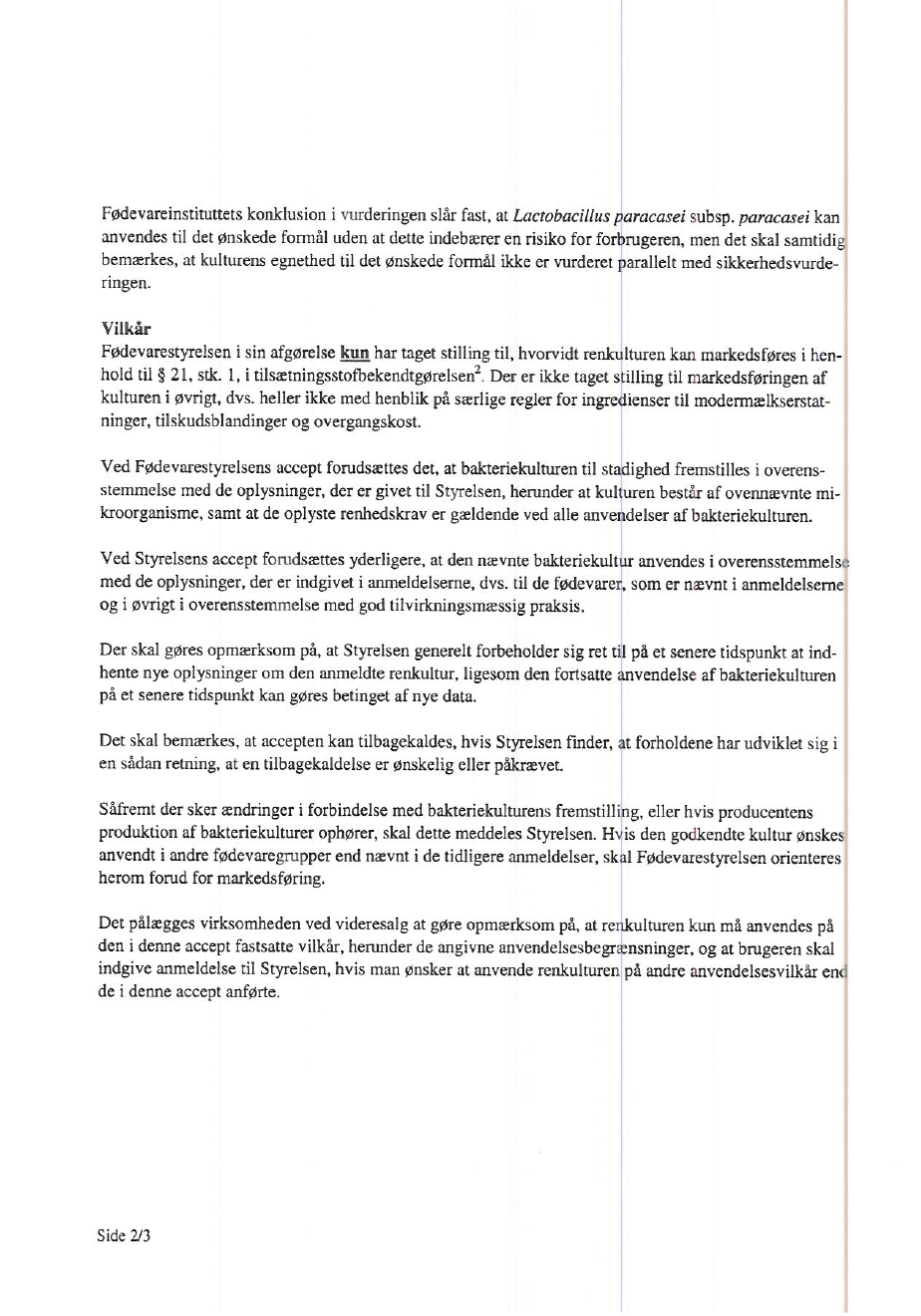


APPENDIX 2. Composition of the infant formulas used in the study.

Table 1. Standard infant formula (arm 1, all formula fed infants before and after intervention and breast-fed infants as spuulement if necessary (<20%)).

| APPROXIMATE COMPOSITION | Unit | Per 100 g powder | Per 100 kJ in formula | 国家标准National standard  (GB10765-2010)  (Unit /100 KJ ) | |
| --- | --- | --- | --- | --- | --- |
|  |  |  |  | 最低Min | 最高Max |
| Macro nutrients大料 | | | | | |
| Energy能量 | kJ | 2130 | 100 |  |  |
| Protein (N x 6,25)蛋白质 | g | 11.3 | 0.53 | 0.45 | 0.7 |
| - Whey Protein乳清蛋白 | g | 6.8 | 0.32 |  |  |
| - Casein酪蛋白 | g | 4.5 | 0.21 |  |  |
| Carbohydrates (total)碳水化合物 | g | 54 | 2.54 | 2.2 | 3.3 |
| FAT/LIPIDS | g | 27.8 | 1.31 | 1.05 | 1.4 |
| - Linoleic acid亚油酸 1) | g | 5.1 | 0.24 | 0.07 | 0.33 |
| - Linoleinic acid亚麻酸 2) | mg | 450 | 21 | 12 | N.S |
| - DHA | mg | 92 | 4.3* | N.S. | 0.5%TFA |
| - ARA | mg | 100 | 4.7** | N.S. | 1%TFA |
| Vitamins and similar nutrients维生素及维生素类营养素 | | | | | |
| Vitamin A | μgRE | 600 | 28 | 14 | 43 |
| Vitamin D | μg | 7.5 | 0.35 | 0.25 | 0.6 |
| Vitamin E | mg α-TE | 10.5 | 0.49 | 0.12 | 1.2 |
| Vitamin K | μg | 42 | 1.97 | 1 | 6.5 |
| Vitamin C | mg | 80 | 3.76 | 2.5 | 17 |
| Vitamin B1 | μg | 580 | 27 | 14 | 72 |
| Vitamin B2 | μg | 1330 | 62 | 19 | 119 |
| Niacin 烟酸 | μg | 4000 | 188 | 70 | 360 |
| Pantothenic acid泛酸 | μg | 3550 | 167 | 96 | 478 |
| Vitamin B6 | μg | 320 | 15 | 8.5 | 45 |
| Biotin生物素 | μg | 18 | 0.85 | 0.4 | 2.4 |
| Folic acid叶酸 | μg | 100 | 4.7 | 2.5 | 12 |
| Vitamin B12 | μg | 2.4 | 0.11 | 0.025 | 0.36 |
| Choline胆碱 | mg | 100 | 4.7 | 1.7 | 12 |
| Inositol肌醇 | mg | 32 | 1.5 | 1 | 9.5 |
| Taurine牛磺酸 | mg | 40 | 1.9 | N.S. | 3 |
| L-Carnitine左旋肉碱 | mg | 10 | 0.47 | 0.3 | N.S. |
| Lutein叶黄素 | μg | 38 | 1.8 | *** | *** |
| Nucleotides核苷酸 | mg | 23 | 1.1 | **** | **** |
| Minerals 矿物质 | | | | | |
| Calcium - Ca 钙 | mg | 400 | 18.8 | 12 | 35 |
| Phosphorus - P磷 | mg | 260 | 12.2 | 6 | 24 |
| Potassium - K钾 | mg | 520 | 24.4 | 14 | 43 |
| Sodium - Na钠 | mg | 145 | 6.8 | 5.0 | 14 |
| Chloride - Cl氯 | mg | 330 | 15.5 | 12 | 38 |
| Magnesium - Mg镁 | mg | 64 | 3.0 | 1.2 | 3.6 |
| Iron - Fe铁 | mg | 6.1 | 0.29 | 0.1 | 0.36 |
| Zinc - Zn锌 | mg | 4.6 | 0.22 | 0.12 | 0.36 |
| Copper - Cu铜 | µg | 430 | 20.2 | 8.5 | 29 |
| Iodide - I碘 | µg | 95 | 4.5 | 2.5 | 14 |
| Manganese - Mn锰 | µg | 68 | 3.2 | 1.2 | 24 |
| Selenium-硒 | µg | 15 | 0.70 | 0.48 | 1.9 |
| 1) The linoleic:α-linolenic acid ratio shall not be less than 5 nor greater than 15. | | | | |  |
| 2) Calcium:phosphorus ratio shall not be less than 1 nor greater than 2 | | | |  |  |

* equals 0,33% of total fatty acids (TFA)

** equals 0,36% of total fatty acids (TFA)

*** Min 300 μg/kg, max 2000 μg/kg (GB 14880)

**** Min 120 mg/kg, max 580 mg/kg (GB 14880)

Table 2. Standard infant formula with *L. paracasei ssp paracasei* strain F19 (arm 2).

| APPROXIMATE COMPOSITION | Unit | Per 100 g powder | Per 100 kJ in formula | 国家标准National standard  (GB10765-2010)  (Unit /100 KJ ) | |
| --- | --- | --- | --- | --- | --- |
|  |  |  |  | 最低Min | 最高Max |
| Macro nutrients大料 | | | | | |
| Energy能量 | kJ | 2130 | 100 |  |  |
| Protein (N x 6,25)蛋白质 | g | 11.3 | 0.53 | 0.45 | 0.7 |
| - Whey Protein乳清蛋白 | g | 6.8 | 0.32 |  |  |
| - Casein酪蛋白 | g | 4.5 | 0.21 |  |  |
| Carbohydrates (total)碳水化合物 | g | 54 | 2.54 | 2.2 | 3.3 |
| FAT/LIPIDS | g | 27.8 | 1.31 | 1.05 | 1.4 |
| - Linoleic acid亚油酸 1) | g | 5.1 | 0.24 | 0.07 | 0.33 |
| - Linoleinic acid亚麻酸 2) | mg | 450 | 21 | 12 | N.S |
| - DHA | mg | 92 | 4.3* | N.S. | 0.5%TFA |
| - ARA | mg | 100 | 4.7** | N.S. | 1%TFA |
| Vitamins and similar nutrients维生素及维生素类营养素 | | | | | |
| Vitamin A | μgRE | 600 | 28 | 14 | 43 |
| Vitamin D | μg | 7.5 | 0.35 | 0.25 | 0.6 |
| Vitamin E | mg α-TE | 10.5 | 0.49 | 0.12 | 1.2 |
| Vitamin K | μg | 42 | 1.97 | 1 | 6.5 |
| Vitamin C | mg | 80 | 3.76 | 2.5 | 17 |
| Vitamin B1 | μg | 580 | 27 | 14 | 72 |
| Vitamin B2 | μg | 1330 | 62 | 19 | 119 |
| Niacin 烟酸 | μg | 4000 | 188 | 70 | 360 |
| Pantothenic acid泛酸 | μg | 3550 | 167 | 96 | 478 |
| Vitamin B6 | μg | 320 | 15 | 8.5 | 45 |
| Biotin生物素 | μg | 18 | 0.85 | 0.4 | 2.4 |
| Folic acid叶酸 | μg | 100 | 4.7 | 2.5 | 12 |
| Vitamin B12 | μg | 2.4 | 0.11 | 0.025 | 0.36 |
| Choline胆碱 | mg | 100 | 4.7 | 1.7 | 12 |
| Inositol肌醇 | mg | 32 | 1.5 | 1 | 9.5 |
| Taurine牛磺酸 | mg | 40 | 1.9 | N.S. | 3 |
| L-Carnitine左旋肉碱 | mg | 10 | 0.47 | 0.3 | N.S. |
| Lutein叶黄素 | μg | 38 | 1.8 | *** | *** |
| Nucleotides核苷酸 | mg | 23 | 1.1 | **** | **** |
| Minerals 矿物质 | | | | | |
| Calcium - Ca 钙 | mg | 400 | 18.8 | 12 | 35 |
| Phosphorus - P磷 | mg | 260 | 12.2 | 6 | 24 |
| Potassium - K钾 | mg | 520 | 24.4 | 14 | 43 |
| Sodium - Na钠 | mg | 145 | 6.8 | 5.0 | 14 |
| Chloride - Cl氯 | mg | 330 | 15.5 | 12 | 38 |
| Magnesium - Mg镁 | mg | 64 | 3.0 | 1.2 | 3.6 |
| Iron - Fe铁 | mg | 6.1 | 0.29 | 0.1 | 0.36 |
| Zinc - Zn锌 | mg | 4.6 | 0.22 | 0.12 | 0.36 |
| Copper - Cu铜 | µg | 430 | 20.2 | 8.5 | 29 |
| Iodide - I碘 | µg | 95 | 4.5 | 2.5 | 14 |
| Manganese - Mn锰 | µg | 68 | 3.2 | 1.2 | 24 |
| Selenium-硒 | µg | 15 | 0.70 | 0.48 | 1.9 |
| 1) The linoleic:α-linolenic acid ratio shall not be less than 5 nor greater than 15. | | | |  |  |
| 2) Calcium:phosphorous ratio shall not be less than 1 nor greater than 2 | | | |  |  |
|  |  |  |  |  |  |
| Probiotics 益生菌指标 | | | | | |
| Lactobacillus paracasei ssp. paracasei strain F19 | Cfu | 1*10^8 *****^ |  |  |  |

* equals 0,33% of total fatty acids (TFA)

** equals 0,36% of total fatty acids (TFA)

*** Min 300 μg/kg, max 2000 μg/kg (GB 14880)

**** Min 120 mg/kg, max 580 mg/kg (GB 14880)

***** as minimum during shelf life

Table 3. Standard infant formula with a WPC (Lacprodan MFGM) (arm 3).

| APPROXIMATE COMPOSITION | Unit | Per 100 g powder | Per 100 kJ in formula | 国家标准National standard  (GB10765-2010)  (Unit /100 KJ ) |  |
| --- | --- | --- | --- | --- | --- |
|  |  |  |  | 最低Min | 最高Max |
| Macro nutrients大料 |  |  |  |  |  |
| Energy能量 | kJ | 2130 | 100 |  |  |
| Protein (N x 6,25)蛋白质 | g | 11.3 | 0.53 | 0.45 | 0.7 |
| - Whey Protein乳清蛋白 | g | 6.8 | 0.32 |  |  |
| - Casein酪蛋白 | g | 4.5 | 0.21 |  |  |
| Carbohydrates (total)碳水化合物 | g | 54 | 2.54 | 2.2 | 3.3 |
| FAT/LIPIDS | g | 27.8 | 1.31 | 1.05 | 1.4 |
| - Linoleic acid亚油酸 1) | g | 5.1 | 0.24 | 0.07 | 0.33 |
| - Linoleinic acid亚麻酸 2) | mg | 440 | 21 | 12 | N.S |
| - DHA | mg | 92 | 4.3* | N.S. | 0.5%TFA |
| - ARA | mg | 100 | 4.7** | N.S. | 1%TFA |
| Vitamins and similar nutrients维生素及维生素类营养素 | | | | |  |
| Vitamin A | μgRE | 600 | 28 | 14 | 43 |
| Vitamin D | μg | 7.5 | 0.35 | 0.25 | 0.6 |
| Vitamin E | mg α-TE | 10 | 0.47 | 0.12 | 1.2 |
| Vitamin K | μg | 42 | 1.97 | 1 | 6.5 |
| Vitamin C | mg | 80 | 3.76 | 2.5 | 17 |
| Vitamin B1 | μg | 580 | 27 | 14 | 72 |
| Vitamin B2 | μg | 1330 | 62 | 19 | 119 |
| Niacin 烟酸 | μg | 4000 | 188 | 70 | 360 |
| Pantothenic acid泛酸 | μg | 3550 | 167 | 96 | 478 |
| Vitamin B6 | μg | 320 | 15 | 8.5 | 45 |
| Biotin生物素 | μg | 17 | 0.80 | 0.4 | 2.4 |
| Folic acid叶酸 | μg | 100 | 4.7 | 2.5 | 12 |
| Vitamin B12 | μg | 2.4 | 0.11 | 0.025 | 0.36 |
| Choline胆碱 | mg | 100 | 4.7 | 1.7 | 12 |
| Inositol肌醇 | mg | 32 | 1.5 | 1 | 9.5 |
| Taurine牛磺酸 | mg | 40 | 1.9 | N.S. | 3 |
| L-Carnitine左旋肉碱 | mg | 10 | 0.47 | 0.3 | N.S. |
| Lutein叶黄素 | μg | 38 | 1.8 | *** | *** |
| Nucleotides核苷酸 | mg | 24 | 1.1 | **** | **** |
| Minerals 矿物质 |  |  |  |  |  |
| Calcium - Ca 钙 | mg | 390 | 18 | 12 | 35 |
| Phosphorus - P磷 | mg | 240 | 11 | 6 | 24 |
| Potassium - K钾 | mg | 480 | 23 | 14 | 43 |
| Sodium - Na钠 | mg | 140 | 6.6 | 5.0 | 14 |
| Chloride - Cl氯 | mg | 330 | 15 | 12 | 38 |
| Magnesium - Mg镁 | mg | 64 | 3.0 | 1.2 | 3.6 |
| Iron - Fe铁 | mg | 6.1 | 0.29 | 0.1 | 0.36 |
| Zinc - Zn锌 | mg | 4.6 | 0.22 | 0.12 | 0.36 |
| Copper - Cu铜 | µg | 430 | 20.2 | 8.5 | 29 |
| Iodide - I碘 | µg | 87 | 4.1 | 2.5 | 14 |
| Manganese - Mn锰 | µg | 68 | 3.2 | 1.2 | 24 |
| Selenium-硒 | µg | 15 | 0.70 | 0.48 | 1.9 |
| 1) The linoleic:α-linolenic acid ratio shall not be less than 5 nor greater than 15. | | | | |  |
| 2) Calcium:phosphorus ratio shall not be less than 1 nor greater than 2 | | | |  |  |

* equals 0,33% of total fatty acids (TFA)

** equals 0,36% of total fatty acids (TFA)

*** Min 300 μg/kg, max 2000 μg/kg (GB 14880)

**** Min 120 mg/kg, max 580 mg/kg (GB 14880)

APPENDIX 3 Summary on safety aspects of Lactobacillus paracasei ssp paracasei F19

Lactic acid bacteria and safety in general

Lactic acid bacteria are part of the human GI-microbiota. They are normally found in numbers from 0.01 million to 100 millions per g faeces or per g/intestinal content in the large intestine. They are also adhering to the mucus and to the intestinal epithelium. Humans have been living together with lactic acid bacteria since the evolution started, as lactobacilli are present in most foods and in high numbers in all fermented, non-heat-treated foods. The daily intake of lactic acid bacteria by food in Scandinavia is at present around 10-100 billions mainly from fermented milk and cheese. Specially selected probiotic bacteria might be 1-10% of total intake. Regarding safety aspects lactobacilli are rarely a causative agent for infections or result in other problems. (EFSA, Borriello et al 2003, Mogensen 2003, Salminen 1998).

Origin of Lactobacillus F19

Lactobacillus F19 is isolated from a sample of human colon (Ljungh et al 2002). Isolates identical with Lactobacillus F19 according to methods described below have been found in a cheese sample and in several samples from humans not being deliberately exposed to Lactobacillus F19 (Björneholm et al 2002, Crittenden et al 2002, Sullivan et al 2002, Sullivan et al 2003). The species Lactobacillus paracasei ssp paracasei is also commonly isolated from food and human GI-microflora (Dunne et al 2001, Molin 2002) By consuming foods with Lactobacillus F19 humans are exposed to bacteria already present in foods and a natural component of the normal GI-microbiota.

Strain identification

Lactobacillus F19 has been identified as a strain belonging to L. paracasei ssp. paracasei by phenotypic typing, SDS-PAGE cluster analysis, Riboprinter, FTIR-analysis and with a 16S RNA probe (Björneholm et al 2002), and by whole genome sequencing using Sanger sequencing technology (Integrated Genomics 2009). The Lactobacillus F19 strain has been part of the EU-PROSAFE project in which the strain was identified as a L. paracasei ssp. paracasei using the molecular methods PFGE and AFPL (Vankerckhove et al 2008).

Genetic stability

Lactobacillus F19 contains three different, small plasmids. Those might change if not handled correct as other genes. The stability of Lactobacillus F19 has been studied and proven not to change during roughly ten years. Plasmids were not lost and were difficult to cure, one resisted all attempts. The antibiotic resistant pattern was not changed due to storage and curing of the plasmids possible to cure (Morelli et al 2002).

Deposition

Lactobacillus F19 is deposited at LMG, Gent, Belgium as LMG P.17806. The strain is freely available to the scientific community according rules following US patent laws – US patent 6,599,504 B1.

In-vitro properties

In-vitro properties might give some indications regarding safety. Some aspects of possible importance are given below but in-vitro properties are summarized in a specific paper (Lactobacillus paracasei ssp paracasei F19 – in vitro investigations, by Fondén R 2004).

Lactic acid: Lactobacillus F19 is exclusively forming L-lactic acid in vitro and in a model of the human GI-tract. This might be of importance as D-lactic acid when formed in high amounts might give acidosis in individuals with a low metabolism of D-lactic acid (Uribarri et al 1998).

Bile acids are deconjugated in the lower ileum. It has been suggested that a deconjugation in the upper parts of ileum might be negative. Lactobacillus F19 do not deconjugate bile acids in-vitro (Anon 1995). Adhesion has been suggested to be a potential property of importance to risk of pathogenicity (Harty et al 1994) Lactobacillus F19 adheres only rather limited to different collagens and to Matrigel. Further it did not aggregate human platelets (Fondén et al 2003).

Antibiotic resistance

The antibiotic resistant pattern of Lactobacillus F19 is known (Table 1). It does not differ from the natural resistance pattern of the L. casei-group. Like all strains belonging to the L. casei-group it is naturally resistant to vancomycin (Charteris et al 1998, Danielsen and Wind 2003, Mändar et al 2001). Further Lactobacillus F19 is sensitive to erythromycin, an antibiotic to which some lactobacilli strains are known to be resistant and which might be transferred to other bacteria (Gevers 2003). Lactobacillus F19 has been given together with clindamycin to healthy volunteers. In spite of the high numbers present of Lactobacillus F19 no strains could be isolated with an increased resistance to clindamycin (Sullivan et al 2003). In another study patients with pneumonia or pyelitis was treated with either penicillin or ciprofloxacin/norfloxacin. No side effects in form of increased antibiotic resistance due to Lactobacillus F19 were noted and resistance to vancomycin of enterococci did not increase (Sullivan et al 2004). Comparison of the genome of Lactobacillus F19 with other sequenced genomes of the L. casei-group shows that Lactobacillus F19 contains genes related to antibiotic resistance at the same extent as the others.

Table 1. Antibiotic resistance pattern of Lactobacillus F19.

| Resistant | Sensitive |
| --- | --- |
| Aztreonam | Penicillin G |
| Ceftaxidime | Ampicillin |
| Cefotaxime | Bacitracin |
| Cefoxitin | Gentamycin |
| Polymyxin B | Clindamycin |
| Vancomycin | Chloramphenicol |
|  | Erythromycin |
|  | Rifampicin |
|  | Streptomycin |
|  | Tetracycline |
|  | Trimetoprim |

Animal studies

Lactobacillus F19 has been given to mice, both ordinary and mice treated with cyclophosphamid, a substance making the mice sensitive to infections (Trautmann et al 1984). In spite of this treatment the mice intake of Lactobacillus F19 did not cause any side effects (Aleljung et al 1999). The same study also showed that the rate of translocation did not increase when mice were given Lactobacillus F19. Lactobacillus F19 has also been given to gnotobiotic mice without any negative impact on health properties. The global gene expression did not reveal any unexpected changed in the gene expression pattern (Nerstedt et al 2007).

Tolerance

Lactobacillus F19 has since 2001 been present in consumer products such as Gaio Dofilus, Gaio Yoghurtdryck, and in products of the Cultura brand Denmark and Sweden. No side effects have been reported. In a surveillance program of clinical blood samples no increase of lactobacilli in such samples has been noted for the period 1998-2004 in comparison to the time period before. The incidence of bacteraemia cases caused by lactobacilli remained at the same level during the study period and constituted <1% of the total number of bacteraemia cases each year. Lactobacillus F19 was not identified in any of the samples (Sullivan et al 2006).

Human studies

Lactobacillus F19 has been given to volunteers of different ages in several clinical trials on infants, healthy adults, elderly and IBS patients. The survival of Lactobacillus F19 through the gastro-intestinal tract has been studied as a part of the FLAIR project (Mörtberg-Backlund et al 1994) and in many of the conducted clinical studies thereafter as a test of compliance and these show that Lactobacillus F19 survives the passage through the gastro-intestinal tract. Most of the studies have been blind and placebo-controlled. As part of a multicentric European project Lactobacillus F19 was included in four pilot feeding studies. The target groups in these studies ranged from infants to elderly and included both healthy subjects and individuals with mild health disorder (milk-hypersensistivity and H. pylori infection). No adverse effects of Lactobacillus F19 administered were observed in any of the pilot studies (Crittenden et al 2002). Lactobacillus F19 has also given to healthy volunteers together with clindamycin and to patients with pneumonia or pyelitis treated with either penicillin or ciprofloxacin/ norfloxacin. No cases of side effects due to Lactobacillus F19 have been reported (Sullivan et al 2001, 2002, 2003, 2004). A symbiotic combination of Lactobacillus F19, other LAB and a fibre has been given to patients with various acute and chronic liver conditions and had no adverse events or adverse changes in general clinical state of the patients (Bengmark 2004). Lactobacillus F19 has been given daily to infants from 4 to 13 months of age with no reported side effects due to Lactobacillus F19 (West at al 2008). Lactobacillus F19 has also been given to IBS patients in two separate studies. Lactobacillus F19 was well tolerated and no adverse events were reported by the patients (Simrén et al 2009, submitted). Finally, Lactobacillus F19 has been given to patients suffering from chronic fatigue syndrome (Sullivan et al 2009), to slightly overweight adults in a study on metabolism and to healthy adults in a study on infection (both yet to be published). No adverse side effects were reported in any of these studies.

References:

Aleljung P, Rupar R and Wadström T, 1999. Rapport om: In vitro och in vivo antagonistisk effect av Lactobacillus spp mot Helicobacter pylori (translokationsexperimenten med F19 och Tyko-projekten är inkluderade). Internal report, Dept of Infectious Diseases and Medical Microbiology, Lund University, Sweden.

Anon, 1995. The selection and characterisation of human probiotic strains, the process technology necessary for their incorporation into fermented foods and evaluation in selected animal models. Final technical report FLAIR AGRF-CT91-0053

Bengmark S, 2004. Synbiotics to strengthen gut barrier function and reduce morbidity in critically ill patients. Clinical Nutrition, vol 23 issue 4: pp 441-445 (editorial)

Björneholm S, Eklöw A, Saarela M and J Mättö, 2002. Enumeration and identification of Lactobacillus paracasei ssp paracasei F19 Microb Ecol Health Dis 2002 Suppl 3: 7-13

Borriello SP, Hammes WP, Holzapfel W, Marteau P, Schrezenmeir J, Vaara M, Valtonen V, 2003. Safety of probiotics that contain lactobacilli or bifidobacteria. Clin Infect Dis. 36:775-80

Charteris WP, Kelly PM, Morelli L, Collins JK, 1998. Antibiotic susceptibility of potentially probiotic Lactobacillus species. J Food Prot. 1998 Dec;61(12):1636-43

Crittenden R, Saarela M, Määtö J , Ouwehand A, Salminen S, Pelto L, Vaughan E, de Vos W, von Wright A, Fondén R and T Mattila-Sandholm, 2002. Lactobacillus paracasei ssp paracasei F19: survival, ecology and safety in the human intestinal tract – a survey of feeding studies within the Probdemo project. Microb Ecol Health Dis 2002 Suppl 3: 22 -26

Danielsen M and A Wind, 2003. Susceptibility of Lactobacillus spp. to antimicrobial agents. Int J Food Microbiol. 82(1):1-11

Dunne C, O´Mahoney L, Murphy L, Thornton G, Morrissey D, O´Halloran S, Feeney M, Flynn S, Fitzgerald G, Daly C, Kiely B, O´Sullivan GC, Shanahan F and JK Collins, 2001. In vitro selection criteria for probiotic bacteria of human origin: correlation with in vivo findings Am J Clin Nutr. 73(2 Suppl):386S-392S.

Fondén R, Björneholm S and K Ohlson, 2003. Lactobacillus F19 – safety considerations in practice. In Fermented Milk pp159-167, IDF Brussels.

Gevers D, Huys G and J Swings, 2003. In vitro conjugal transfer of tetracycline resistance from Lactobacillus isolates to other Gram-positive bacteria.

FEMS Microbiol Lett. 225:125-30.

Harty DW, Oakey HJ, Patrikakis M, Hume EB, Knox KW, 1994. Pathogenic potential of lactobacilli. Int J Food Microbiol. 24:179-89

Ljung Å, Lan J and N Yanagisawa, 2002. Isolation, selection and characteristics of Lactobacillus paracasei ssp paracasei F19 Microb Ecol Health Dis 2002 Suppl 3: 4-7

Mändar R, Loivukene K, Hutt P, Karki T and M Mikelsaar, 2001. Antibacterial susceptibility of intestinal lactobacilli of healthy children. Scand J Infect Dis. 33:344-9

Molin G, 2002. Probiotics in foods not containing milk or milk constituents, with special reference to Lactobacillus plantarum 299v. Am J Clin Nutr. 73(2 Suppl):380S-385S.

Mogensen G, 2003. Safety aspects of fermented products . In Fermented Milk pp159-167, IDF Brussels.

Morelli L and E Campominosi, 2002. Genetic stability of Lactobacillus paracasei ssp paracasei F19 Microb Ecol Health Dis 2002 Suppl 3: 14-16

M. Mörtberg-Backlund and R. Fondén, 1994. Studies of survival of Lactobacillus casei F19 after passage through the human G.I. tract. Extract of report from Arla (Panova Partner) to FLAIR committee. Rewritten in the Final Technical Report FLAIR AGRF-CT91-0053 (page 34: Results related to objective 5).

Nerstedt A, Nilsson EC, Ohlson K, Håkansson J, Svensson LT, Löwenadler B, Svensson U, Mahlapuu M. Administration of Lactobacillus evokes coordinated changes in the intestinal expression profile of genes regulating energy homeostasis and immune phenotype in mice. British Journal of Nutrition (2007), page 1 - 11

Salminen S, von Wright A, Morelli L, Marteau P, Brassart D, de Vos W, Fondén R, Saxelin M, Collins K and Mogensen G, 1998. Demonstration of safety of probiotics-a review. Int. J. Foodmicrob Oct 20, 1998. v. 44 (1/2): 93-106

Sullivan Å, Palmgren AC, Nord E, 2001. Effect of Lactobacillus paracasei on intestinal colonization of lactobacilli, bifidobacteria and C. difficile in elderly persons. Anaerobe (2001) 07:67-70

Sullivan Å, Bennet R, Viitanen M, Palmgren A-C and CE Nord, 2002. Influence of Lactobacillus F19 on intestinal microflora in children and elderly persons and impact on Helicobacter pylori infections Microb Ecol Health Dis 2002 Suppl 3: 17-21

Sullivan, Å, Barkholt, L & Nord, C. E., 2003. Lactobacillus acidophilus, Bifidobacterium lactis and Lactobacillus F19 prevent antibiotic-associated ecological disturbances of Bacteroides fragilis in the intestine. J Antimicrob Chemother 52, 308-311.

Sullivan Å, Johansson A, Svenungsson B and CE Nord, 2004. Effect of Lactobacillus F19 on the Emergence of Antibiotic-Resistant Microorganisms in the Intestinal Microflora. JAC 54:791-797

Sullivan Å, Nord CE. Probiotic lactobacilli and bacteraemia in Stockholm. Scandinavian Journal of Infectious Diseases, 2006; 38: 327-331

Sullivan Å, Nord CE, Evengård B. Effect of supplement with lactic-acid producing bacteria on fatigue and physical activity in patients with chronic fatigue syndrome. Nutr J. 2009 Jan 26;8:4.

Trautmann M, Fischer GI, Hahn H, 1984. ( Therapeutic and opsonizing effect of specific antibacterial immunoglobulins in experimental Klebsiella septicemia in mice]- translation from german) Immun Infekt. 12:56-60

Uribarri J, Oh MS, Carroll HJ, 1998. D-lactic acidosis. A review of clinical presentation, biochemical features, and pathophysiologic mechanisms. Medicine (Baltimore). 77:73-82.

Vankerckhovena V, Huysb G, Vancanneytc M, Vaela C, Klared I, Romonde M-B, Entenzaf, JM, Moreillonf P, Windg, RD, Knolg J, Wiertzh E, Pot B, Vaughanj E, Kahlmeterl G, Goossens H. 2008. Biosafety assessment

of probiotics used for human consumption: recommendations from the EU-PROSAFE project. Trends in Food Science & Technology 19 (2008) 102-114

West CE, Gothefors L, GranströmM, Käyhty H, Hammarström M-L, Hernell O. Effects of feeding probiotics during weaning on infections and antibody responses to diphtheria, tetanus and Hib vaccines. Pediatr Allergy Immunol 2008: 19: 53–60.

APPENDIX 4 Summary on safety aspects of the WPC (Lacprodan**®** MFGM)

MFGM in general

The milk fat globule membrane (MFGM) is the membrane surrounding lipid droplets (milk fat) during their secretion in the alveolar lumen of the lactating mammary gland.

In many aspects the nutritional compostion between human and bovine milk differs. However, several proteins associated with the MFGM are highly conserved, with 98-100% of their amino-acid sequences unchanged for 160 million years (since evolution of a common mammalian ancestor) (Lemay et al. 2009).

The MFGM contains proteins and lipids of nutritional and biological importance with documented health benefits (Spitzberg et al. 2005). Some of the proteins present in the MFGM with well-documented biological activities include; MUC-1, XDH/XO (Xanthine dehydrogenase/oxidase), MUC-15, Lactadherin (PAS6/7), Lactoferrin and Butyrophilin. Eventhough, the MFGM proteins represents only 1-4% of total milk protein content, they have continously been reported to play important roles in various cellular processess and defense mechanisms in the newborn mammal (Cavaletto et al. 2008). Several MFGM proteins associated with the phospholipid membrane have documented anti-pathogenic effects and are known to stimulate intestinal maturation (Kvistgaard et al. 2004, Bojesen, 2007, Ignaki, 2010).

Besides the protein components, MFGM also contains several phospholipids with important biological effects (spingomyelin, phosphatidyl choline, phosphatidyl ethanolamine). The phospholipids that are the main building blocks of MFGM have similar profiles in both bovine and human milk (Kynast, 1988). In general, phospholipids, including milk-derived, affect numerous cell functions including growth and development, molecular transport systems, absorption processes, cognition, and myelination in the central nervous system. Another highly active components in MFGM include gangliosides, which are also present in body tissues, particularly the brain. Ganglioside levels are greater in the brain tissue of breast-fed infants than in the brains of formula-fed infants, which suggests that they play a role in neuro development (Oshida et al. 2003, McJarrow et al.2009). Gangliosides are also thought to improve intestinal immune maturation and microflora (Bu *et al.* 2007).

MFGM and safety

Because, humans are naturally exposed to MFGM via dietary intake of various dairy products and due to the high evolutionary conservation of MFGM component in mammals, the major concern regaring the safety aspect of enriching infant formulas with MFGM are primarily of microbiological origin. MFGM must be frationated from milk that has been exposed to contamination of bacteria from both the cows udder and the external environment during proccesing.

Infant clinical studies with bovine MFGM

The majority of infant milk formulas is made from skim milk powder and whey protein concentrate and consequently does not contain any significant MFGM. Hence infant formulas are practically devoid of the biologically important MFGM proteins and lipids. However, nowadays, milk fractions enriched in MFGM have become available on a large scale commercially, and can therefore be added to infant formulas.

Recently the concept of MFGM having a beneficial effect on infectious diseases in infants was tested.

A randomized, double-blind controlled design with 550 infants were conducted to study the efficacy of the milk fat globule membrane (MFGM) enriched protein fraction, WPC (Lacprodan MFGM), in a complementary food, on diarrhea, anemia and micronutrient status (Zavaleta et al. 2011).

The children (n=550), 6 to 11 months old, consumed for 6 months, an instant complementary food fortified with one RDA of multiple micronutrients with the protein source being either the WPC (Lacprodan MFGM) enriched protein fraction or skim milk powder.

The complementary food (40 g) was divied into two servings per day. The nutrient content of the daily complementary food was: energy 192 kcal, protein 6 g, carbohydrate 20 g, fat 9.6 g, calcium 240 mg. The instant complementary fortified food was prepared by Arla Foods Ingredients amba and provided 1 RDA of iron (10 mg) as sulfate, zinc (5 mg as sulfate, copper (0.6 mg) as sulfate, folate (35 mg), vitamin A (375 ug) as retinyl palmitate, thiamin (400 ug), riboflavin (500 ug), vitamin B_12_ (0.5 ug), and ascorbic acid (35 mg) (24).

Results from the study revealed no significant differences in anthropometric parameters between the different groups of children, demonstrating the WPC (Lacprodan MFGM) as a nutritionally valuable whey fraction. The global prevalence of diarrhea was 3.84% and 4.37% in the MFGM group and control group, respectively (P< 0.05). In addition, consumption of the MFGM protein fraction reduced episodes of bloody diarrhea.

The authors concluded, that addition of an MFGM enriched protein fraction to complementary food had beneficial effects on diarrhea in infants and may thus help to improve the health of vulnerable populations.

Another, clinical trial, stil running, is evaluating effects of a MFGM – enriched infant formula with reduced energy and protein content on growth and development (http://clinicaltrials.gov/ct2/show/NCT00624689).

Term infants with birth weight 2500-4500 g that has been fed with breastmilk only or infant formula only before 2 months of age, are included and outcome measures of body composition, glucose tolerance, visual acuity and neurological development are assessed.

The investigators' hypothesis is; that a modified infant formula with reduced energy and protein content and enrichment with bovine Milk Fat Globule Membrane (MFGM) containing bioactive proteins and phospholipids will reduce the ever existing difference in growth and development between formula-fed and breast-fed infants.

Arla foods Ingredients and the WPC (Lacprodan® MFGM)

About Arla foods Ingredients

Arla Food Ingredients provide milk derived ingredients for various application areas, including infant nutrition. Arla Foods have stringent safety and quality standards to maintain product safety throughout the production chain. Certified quality and HACCP asssurance system based on the ISO 22000:2005, ISO 900 standards ensures that all ingredients are of high quality and is produced using processes of the highest standards. The quality system ensures traceability of the products throughout production from raw material to packaging. All ingredients are derived from milk supplied by audited dairy farmers, who are required by law to meet the tough demands with regard to the health and welfare of the dairy cattle and the environment.

WPC (Lacprodan® MFGM)

WPC (Lacprodan® MFGM) is a naturally based whey protein concentrate enriched with particles components of milk fat globular membrane. The raw material used for its production is raw bovine milk that has been delivered to and processed by dairies in Arla Foods Amba in compliance with the requirements stated in The European Commision Counsil Regulation (EC) No 853/2004.

Product Specifications and Statement on Food Safety and Quality Assurance

References

Spitsberg VL. Bovine milk fat globule membrane as a potential nutraceutical (2005). J Dairy Sci;88:2289-94.

Zavaleta N, Kvistgaard AS, Graverholt G, Respicio G, Guija H, Valencia N, Lönnerdal B.(2011) Efficacy of a Complementary Food Enriched with a Milk Fat Globule Membrane Protein Fraction on Diarrhea, Anemia and Micronutrient Status in Infants. J Pediatr Gastroenterol Nutr. [Epub ahead of print]

Cavaletto M, Giuffrida MG, Conti A. Milk fat globule membrane components - a proteomic approach

Adv Exp Med Biol. 2008;606:129-41.

Kvistgaard A. S. et al. (2004). Inhibitory effects of human bovine milk constituents on rotavirus infections. J. American Dairy Science Association 87: 4088-96

Inagaki et al. (2010). The bovien Lactophorin C-terminal fragment and PAS6/7 were both potent in the inhibition of human rotavirus replication in cultured epithelial cells and the prevention of experimental gastroenteritis. Biosci. Biotechnol. Biochem., 74 (7): 1386-90

Kynast G. (1988). Determination of the phospholipid content of human milk, cow's milk and various infant formulas. Z Ernarungswiss Dec; 27(4): 252-65

Bojesen A. et al. (2007). Inhibitory activities of bovine macromolecular whey proteins on rotavirus infections in vitro and in vivo. J. Dairy Sci. 90: 66-74

Bu H. et al. (2007). Milk fat globule-EGF factor 8/lactadherin plays a crutial role in maintenance and repair of murine intestinal epithelium. The journal of clin invest. 117. 12: 3673-83

McJarrow P. et al. (2009). Influence of dietary gangliosides on neonatal brain development. Nutrition Reviews Vol. 67(8): 451– 63.

Oshida k. et al. (2003). Effects of Dietary Sphingomyelin on Central Nervous System Myelination in Developing Rats. Pediatr Res 53: 589–93

APPENDIX 5 SCORAD evaluation sheet

APPENDIX 6 Adverse events form

| Subject N°: |__|__|__| | Subject initials: |__|__|__|__| |  |
| --- | --- | --- |

| N° AE | Description of AE | Date of start  Day Month Year | Date of end  Day Month Year | Grading^**^ | Measures taken, incl. medication, dosage or discontinuation of study product |
| --- | --- | --- | --- | --- | --- |
| 1 |  | |__|__|__|__|__|__|__| | |__|__|__|__|__|__|__| | Intensity (1-3) ___  Frequency (1-3 or 9) ___  Relation to study product (1-4) ___ |  |
| 2 |  | |__|__|__|__|__|__|__| | |__|__|__|__|__|__|__| | Intensity (1-3) ___  Frequency (1-3 or 9) ___  Relation to study product (1-4)___ |  |
| 3 |  | |__|__|__|__|__|__|__| | |__|__|__|__|__|__|__| | Intensity (1-3) ___  Frequency (1-3 or 9) ___  Relation to study product (1-4) ___ |  |
| 4 |  | |__|__|__|__|__|__|__| | |__|__|__|__|__|__|__| | Intensity (1-3) ___  Frequency (1-3 or 9) ___  Relation to study product (1-4) ___ |  |
| 5 |  | |__|__|__|__|__|__|__| | |__|__|__|__|__|__|__| | Intensity (1-3) ___  Frequency (1-3 or 9) ___  Relation to study product (1-4) ___ |  |
| 6 |  | |__|__|__|__|__|__|__| | |__|__|__|__|__|__|__| | Intensity (1-3) ___  Frequency (1-3 or 9) ___  Relation to study product (1-4) ___ |  |

Intensity: light=1; moderate=2; severe=3

Frequency : rare=1; quite frequent=2; often=3 ; non applicable=9

Relation to study product : unrelated=1; unlikely=2; probable=3; definitely related=4

APPENDIX 7 Serious adverse events form

| Form for Registration of Serious Adverse Event (SAE)  Subject N°: |__|__|__| | | | | | | | |
| --- | --- | --- | --- | --- | --- | --- | --- |
| Initial  Follow-up  Final | | | | | | | |
| Date of birth: **|**__|__**|**__|__|__**|**__|__**|** Current weight (kg): **|**__|__**| .** |__| Sex: M  F  Day Month Year  Date of first study product administration: **|**__|__**|**__|__|__**|**__|__**|**  Day Month Year | | | | | | | |
| Date of onset of SAE: **|**__|__**|**__|__|__**|**__|__**|**  Day Month Year  Diagnosis/symptoms:      Date of resolution: **|**__|__**|**__|__|__**|**__|__**|**  Day Month Year | | | | | | | |
| Seriousness | | | | Clinical course | | Causal relationship  (investigator’s opinion) | |
| Subject died  Hospitalization needed/prolonged  Persistent or significant disability  Congenital anomaly/birth defect  Medically relevant event | | | | Persistent event  Improving  Recovered  Recovered with sequelae  Worsening  Death  Unknown | | Unrelated  Unlikely  Probable  Certain | |
| Previous adverse reaction to similar treatment: Yes  No  Unknown  If yes, give details:  Measures taken concerning the study product:  None  Withdrawal of study product  → date of withdrawal: **|**__|__**|**__|__|__**|**__|__**|**  Day Month Year  Other, specify: | | | | | | | |
|  |  | | | | | | |
| Code breaking: Yes  No  Not applicable  If yes, product code or name: ____________________ Batch No: ______________________ | | | | | | | |
| Concomitant medication and SAE treatment: | | | | | | | |
| Name | | Indication | Route of administration | | Dose  (units) | Dates  Started Ended | |
|  | |  |  | |  |  |  |
|  | |  |  | |  |  |  |
|  | |  |  | |  |  |  |
|  | |  |  | |  |  |  |
|  | |  |  | |  |  |  |
|  | |  |  | |  |  |  |
| If further examinations are required to assess causality, note findings and attach results of examinations (lab reports, X-rays, etc): | | | | | | | |
| Other medical comments: | | | | | | | |
| Principal Investigator (or Co-Investigator):    Printed name Signature Date | | | | | | | |

APPENDIX 8 Dietary and health journal

Subject initials________________

ID code: ________

Day from 00 to 24 hrs (1-3): ________ Date: ________

| Time | Infant formula/ foods/drink  Stool consistency | Amount + unit |  | Data  Code | Data  Code |
| --- | --- | --- | --- | --- | --- |
|  |  |  |  |  |
|  |  |  |  |  |
|  |  |  |  |  |
|  |  |  |  |  |
|  |  |  |  |  |
|  |  |  |  |  |
|  |  |  |  |  |
|  |  |  |  |  |
|  |  |  |  |  |
|  |  |  |  |  |
|  |  |  |  |  |
|  |  |  |  |  |
|  |  |  |  |  |
|  |  |  |  |  |

APPENDIX 9. Parents/subject’s legal representatives’ study information and informed consent form.
